# Differences in outcomes among patients with atrial fibrillation undergoing catheter ablation with versus without intracardiac echocardiography

**DOI:** 10.1111/jce.15599

**Published:** 2022-07-23

**Authors:** Rhea C. Pimentel, Neloufar Rahai, Sonia Maccioni, Rahul Khanna

**Affiliations:** ^1^ Department of Cardiovascular Medicine The University of Kansas Health System Kansas City Kansas USA; ^2^ Medical Device Epidemiology and Real‐World Data Sciences Johnson & Johnson New Brunswick New Jersey USA; ^3^ Franchise Health Economics and Market Access Johnson & Johnson Irvine California USA

**Keywords:** acute ischemic stroke, atrial fibrillation, catheter ablation, intracardiac echocardiography

## Abstract

**Background:**

Intracardiac echocardiography (ICE) use can lead to early detection of periprocedural complications and may improve patient outcomes by providing real‐time visualization of catheter location and the treatment area during cardiac ablation (CA) for atrial fibrillation (AF).

**Objective:**

Examine complications and 12‐month healthcare use among patients with AF undergoing CA with versus without ICE use during the procedure in a real‐world setting.

**Methods:**

The 2015–2020 IBM MarketScan® Database was used to identify non‐elderly adults (age 18–64 years) undergoing CA for AF. Patients were classified into ICE/non‐ICE groups based on the presence or absence of ICE procedure codes. Patients in each group were matched on study covariates using propensity scores. Peri‐procedural complications, 12‐month cardiovascular (CV) or AF‐related inpatient admission, repeat CA, and cardioversion were compared using a Cox proportional hazard model.

**Results:**

1371 patients were identified in each study cohort (ICE and non‐ICE) after propensity matching. Patients who had CA with ICE had a significantly lower rate of complications than those without (2.9% vs. 5.8%; *p* < .001). The risk of complications was 50% lower with ICE use (hazard ratio [HR] 0.50; 95% confidence interval [CI] 0.34–0.72). For assessment of 12‐month healthcare utilization, 1250 patients were identified in each cohort after propensity matching. ICE use was associated with a 36% lower risk of 12‐month repeat ablation (HR 0.64; 95% CI 0.49–0.83). No differences in CV‐ or AF‐related inpatient admission and cardioversion were observed.

**Conclusion:**

Among patients with AF, the use of ICE during an ablation procedure was associated with lower incidence of complications and repeat ablation.

**Abbreviations**
AAD: antiarrhythmic drug
AF: atrial fibrillation
CA: catheter ablation
CDHP: consumer directed health plan
CHADS2VASc: stands for congestive heart failure, hypertension, age≥75 years, diabetes mellitus, stroke or transient ischemic attack, vascular disease, age 65 to 74 years, sex category
CI: confidence interval
CV: cardiovascular
EPO: exclusive provider organization
HDHP: high deductible health plan
HMO: health maintenance organization
HR: hazard ratio
ICE: intracardiac echocardiography
IRB: Institutional Review Board
POS: point of service
PPO: preferred provider organization
SMD: standardized mean difference
US: United States

## 1 INTRODUCTION

The incidence of atrial fibrillation (AF) continues to rise as the overall population ages.^1^ By the middle of the century, the number of individuals with AF is expected to reach 14 million in Europe,^2^ and as high as 16 million in the United States (US).^3^ The burden of AF in the Asia‐Pacific region is projected to be particularly high with an estimated 72 million patients by 2050.^4^ Timely management and treatment of patients with AF using safe and effective approaches are critical in alleviating the associated burden of disease.

Recommended treatments for AF include antiarrhythmic drugs (AADs), and, for those who are refractory or intolerant to AADs, catheter ablation (CA).^5^, ^6^, ^7^, ^8^, ^9^, ^10^ Studies have shown that CA may reduce long‐term AF recurrence and can be associated with clinically meaningful improvement in symptoms and quality of life.^11^, ^12^, ^13^, ^14^ Intracardiac echocardiography (ICE) provides high‐resolution real‐time visualization of cardiac structures, continuous monitoring of catheter location, and early recognition of procedural complications, such as pericardial effusion, thrombus formation, or cardiac perforation during CA.^15^, ^16^ Studies have previously suggested that CA outcomes may be further improved with the use of ICE.^16^, ^17^, ^18^, ^19^, ^20^, ^21^, ^22^, ^23^, ^24^, ^25^, ^26^ In one such report, patients with AF who underwent an ablation procedure without ICE had five times higher risk of cardiac perforation compared to those who had the procedure with ICE.^16^

Though studies have demonstrated the incremental safety benefit associated with ICE use, a comprehensive assessment of both long‐term (12‐month) outcomes and safety end points in a large real‐world sample of patients are lacking. To address this gap in the literature, we performed analysis of a nationally representative administrative claims database to evaluate differences in complications and 12‐month AF‐related healthcare use among AF patients undergoing CA with versus without ICE in a contemporary cohort.

## 2 METHODS

### 2.1 Data source

The 2015–2020 IBM MarketScan® Commercial Database,^26^ which is a nationally representative medical and prescription drug insurance claims database for more than 138 million people with primary coverage through privately insured health plans in the US, was used for study purposes. The database includes information on inpatient admissions, outpatient services, prescription drugs, enrollment, and costs associated with healthcare services. The New England Institutional Review Board (IRB) determined that studies conducted in IBM MarketScan® are exempt from study‐specific IRB review as these studies do not involve active human subject participation.

### 2.2 Study population

Patients aged 18–64 years old undergoing CA for AF treatment were identified, with first such procedure considered as index procedure. Patients were required to be continuously enrolled for 12 months before the index procedure. Patients were excluded if, in the 12‐month pre‐index period, they had a CA procedure with a primary or secondary diagnosis of AF, had surgical ablation, left atrial appendage occlusion, valvular procedure, or atrioventricular node ablation.

### 2.3 Study variables

The main independent variable of interest was the use of ICE during CA procedure defined by the presence or absence of ICE procedure codes during index ablation admission. Other study covariates included: age; sex; Elixhauser comorbidity score^28^; CHA₂DS₂‐VASc score^29^; sleep apnea; AAD use (amiodarone, dofetilide, flecainide, propafenone, ibutilide, sotalol, dronedarone, quinidine, and disopyramide); anticoagulant use (warfarin, dabigatran, apixaban, rivaroxaban, edoxaban, betrixaban); geographic region (Northeast, North Central, South, West, and Unknown); insurance plan type (basic or comprehensive plan, consumer‐directed health plan/high deductible health plan, exclusive provider organization/health maintenance organization, point of service plan, preferred provider plan, or unknown insurance type); and procedure year (2016–2020).

### 2.4 Study outcomes

Complications (assessed among patients with index ablation between 2016 and 2020), including acute coronary syndrome, ischemic stroke, hemorrhagic stroke, thromboembolism, transient ischemic attack, phrenic nerve damage, pericarditis, bleeding, vascular access complications, blood transfusion, laryngospasm, mandibular joint dislocation, vagal nerve injury, were assessed within 7 days post‐discharge. Cardiac perforation was assessed within 30 days and atrioesophageal fistula and severe pulmonary vein stenosis within 90 days. A composite of all complications was also included. Appendix B lists the codes used to assess complications.

Healthcare utilization was assessed within 1 year of CA (among patients with index ablation between 2016 and 2019 [to allow 12‐month follow‐up]). Utilization was assessed in the full 12 months after CA and also in the 4–12 months after CA following a standardized 90‐day “blanking period” to avoid capturing early recurrences per Heart Rhythm Society guidelines.^6^ Healthcare utilization outcomes included: AF‐related readmission, cardiovascular (CV)‐related readmission, repeat CA, or electrical cardioversion. Appendix A lists the codes considered for study attrition and outcomes.

### 2.5 Statistical analysis

Bivariate tests comparing the ICE and non‐ICE groups on study variables were performed using Chi‐square tests of independence for categorical variables and analysis of variance tests for continuous variables. Patients who underwent CA with versus without ICE were matched on study covariates using propensity scores. Multivariable logistic regression was used to fit the propensity score model. Balance of study covariates was assessed using standardized mean difference (SMD). Time‐to‐event analyses were used, with patients right‐censored if they died, were lost to follow‐up (defined as gap of >30 days in enrollment), or reached the end of follow‐up time without an outcome of interest. Hazard ratios (HRs) from Cox regression models were used to assess the relationship between ICE use and study outcomes.

The threshold for statistical significance was a *p*‐value < .05. We performed multiplicity adjustment by controlling for the false discovery rate using the Benjamini–Hochberg (BH) procedure. All analyses were conducted using R for Windows, version 4.0.2.^30^

## 3 RESULTS

### 3.1 Complications

A total of 19 368 patients met the criteria for the assessment of complications, with 17 997 (92.9%) in the ICE group and 1371 (7.1%) in the non‐ICE group. Supporting Information: Table S1 shows the study population attrition for the cohort. Table 1 lists the patient baseline characteristics for the ICE and non‐ICE groups before and after propensity score matching. Around a third of patients in both groups (pre‐ and post‐matching) were aged 61 years and above, and approximately 26% were female. Before matching, a significantly higher proportion of patients in the ICE cohort had sleep apnea as compared to those in the non‐ICE cohort (41.5% vs. 35.7%, *p* < .001, SMD = 0.120); however, after matching, no significant difference was observed (35.7% vs. 36.0%, *p* = .905, SMD = 0.006). A significantly higher proportion of patients in the ICE cohort had prior AAD (64.9% vs. 51.0%, *p* < .001, SMD = 0.277) and anti‐coagulant use (77.7% vs. 66.6%, *p* < .001, SMD = 0.235) in the pre‐match cohort. After matching, no significant differences were observed among the ICE and non‐ICE cohorts on prior AAD (51.0% vs. 50.8%, *p* = .939, SMD = −0.004) and anti‐coagulant use (66.6% vs. 68.5%, *p* = .308, SMD = 0.040). Overall, no significant differences on study covariates were observed among the ICE and non‐ICE cohort after propensity matching.

**Table 1:** Baseline characteristics of atrial fibrillation patients pre‐and post‐matching in the complications cohort

	Pre‐match				Post‐match (1:1)			
	ICE, *N* = 17 997	No ICE, *N* = 1371	*p* value	SMD	ICE, *N* = 1371	No ICE, *N* = 1371	*p* value	SMD
**Age**			.032				.973	
18–54	5767 (32.0)	449 (32.7)		.015	449 (32.7)	449 (32.7)		.000
55–60	6612 (36.7)	458 (33.4)		.071	458 (33.4)	453 (33.0)		−.008
61+	5618 (31.2)	464 (33.8)		.056	464 (33.8)	469 (34.2)		.008
**Female**	4811 (26.7)	358 (26.1)	.639	.014	358 (26.1)	329 (24.0)	.217	−.048
**Elixhauser Score 4**+	9767 (54.3)	719 (52.4)	.200	.037	719 (52.4)	723 (52.7)	.909	.006
**CHADS2VASc score 2**+	9461 (52.6)	726 (53.0)	.805	.008	726 (53.0)	739 (53.9)	.646	.019
**Sleep apnea**	7464 (41.5)	490 (35.7)	<.001	.120	490 (35.7)	494 (36.0)	.905	.006
**AAD use**	11 672 (64.9)	699 (51.0)	<.001	.277	699 (51.0)	696 (50.8)	.939	−.004
**Anticoagulant use**	13 979 (77.7)	913 (66.6)	<.001	.235	913 (66.6)	939 (68.5)	.308	.040
**Region**			<.001				.827	
Northeast	2750 (15.3)	408 (29.8)			408 (29.8)	403 (29.4)		−.008
North Central	4002 (22.2)	153 (11.2)		.352	153 (11.2)	139 (10.1)		−.032
South	8736 (48.5)	575 (41.9)		.134	575 (41.9)	593 (43.3)		.027
West	2482 (13.8)	230 (16.8)		.080	230 (16.8)	233 (17.0)		.006
Unknown	27 (0.2)	5 (0.4)		.036	5 (0.4)	3 (0.2)		−.024
**Insurance**			.033				.226	
Basic or comprehensive	616 (3.4)	52 (3.8)		.019	52 (3.8)	39 (2.8)		−.050
CDHP/HDHP	3766 (20.9)	261 (19.0)		.048	261 (19.0)	270 (19.7)		.017
EPO/HMO	1974 (11.0)	178 (13.0)		.060	178 (13.0)	162 (11.8)		−.035
POS	1272 (7.1)	114 (8.3)		.045	114 (8.3)	101 (7.4)		−.034
PPO	10 047 (55.8)	748 (54.6)		.025	748 (54.6)	789 (57.5)		.060
Unknown	322 (1.8)	18 (1.3)		.042	18 (1.3)	10 (0.7)		−.051
**Year**			<.001				.579	
2016	3913 (21.7)	416 (30.3)		.187	416 (30.3)	410 (29.9)		−.010
2017	3799 (21.1)	318 (23.2)		.049	318 (23.2)	343 (25.0)		.043
2018	3956 (22.0)	278 (20.3)		.042	278 (20.3)	274 (20.0)		−.007
2019	3609 (20.1)	238 (17.4)		.071	238 (17.4)	243 (17.7)		.010

*Note*: Data are *n* (%).

Abbreviations: AAD, antiarrhythmic drug use; CDHP, consumer‐directed health plan; CHADS2VASc, stands for congestive heart failure, hypertension, age ≥ 75 years, diabetes mellitus, stroke or transient ischemic attack, vascular disease, age 65–74 years, sex category; EPO, exclusive provider organization; HDHP, high deductible health plan; HMO, health maintenance organization; ICE, intracardiac echocardiography; POS, point of service; PPO, preferred provider organization; SMD, standardized mean difference.

Patients who underwent CA with ICE were less likely to experience acute coronary syndrome in the 7‐day period after the procedure compared to those who had CA without ICE (0.7% vs. 2.8%, *p* < .001). The two groups did not differ significantly for any other complication. Overall, patients who had CA with ICE had a significantly lower rate of composite complication compared to those who had CA without ICE (2.9% vs. 5.8%, *p* < .001). Results from regression analysis showed that ICE patients had a 50% lower risk of composite complication end point compared to non‐ICE patients (HR 0.50, 95% CI 0.34–0.72, log‐rank *p* < .001). Table 2 lists the complication results for both groups.

**Table 2:** Complications among atrial fibrillation patients in the 90 days after catheter ablation by intracardiac echocardiography (ICE) use

	ICE, *N* = 1371	No ICE, *N* = 1371	*p* value	BH adjusted critical value^a^
**Composite complication**	40 (2.9)	80 (5.8)	<.001^b^	**.011**
**Complications within 7 days**				
Acute coronary syndrome	10 (0.7)	38 (1.8)	<.001^b^	**.024**
Ischemic stroke	7 (0.5)	4 (0.3)	.548	.094
Hemorrhagic stroke	1 (0.1)	1 (0.1)	1.000	.118
Thromboembolism	4 (0.3)	10 (0.7)	.178	.035
Transient ischemic stroke	3 (0.2)	4 (0.3)	1.000	.129
Phrenic nerve damage	2 (0.2)	5 (0.4)	.453	.071
Pericarditis	4 (0.3)	6 (0.4)	.754	.106
Bleeding	9 (0.7)	6 (0.4)	.437^b^	.059
Vascular access complications	2 (0.2)	0 (0)	.500	.082
Blood transfusion	1 (0.1)	1 (0.1)	1.000	.141
Laryngospasm	0 (0)	0 (0)	n/a	n/a
Mandibular joint dislocation	0 (0)	0 (0)	n/a	n/a
Vagal nerve injury	0 (0)	0 (0)	n/a	n/a
**Complications within 30 days**				
Cardiac perforation	4 (0.3)	9 (0.7)	.266	.047
**Complications within 90 days**				
Atrioesophageal fistula	1 (0.1)	0 (0)	1.000	.153
Severe pulmonary vein stenosis	0 (0)	0 (0)	n/a	n/a

*Note*: Data are *n* (%).

Abbreviations: BH, Benjamini–Hochberg; CI, confidence interval.

^a^ Benjamini–Hochberg adjusted critical value after correction for multiple comparisons (total number of tests considered for adjustment = 17; false discovery rate of 20%). After BH adjustment, the largest *p* value which was lower than the BH adjusted critical value was identified. We then designated every *p* value smaller than this *p* value as significant. The adjusted values that are bolded represent significant results.

^b^ Chi‐square was used for statistical significance testing; for all other outcomes with cell sizes less than 5, Fisher's exact test was used.

### 3.2 AF‐related healthcare utilization

A total of 16 524 patients met the criteria for the assessment of 12‐month healthcare utilization, with 15 274 (92.4%) in the ICE group and 1250 (7.6%) in the non‐ICE group. Table S2 shows the study population attrition for this cohort. Table 3 lists the patient baseline characteristics for both groups before and after propensity score matching. In the pre‐match cohort, significant differences were observed among the ICE and non‐ICE cohorts on sleep apnea, AAD use, anti‐coagulant use, region, and year of procedure. However, after matching, no significant differences were observed among the ICE and non‐ICE cohort on any study covariate.

**Table 3:** Baseline characteristics of atrial fibrillation patients pre‐and post‐matching in the 12‐month healthcare utilization cohort

	Pre‐match				Post‐match (1:1)			
	ICE, *N* = 15 274	No ICE, *N* = 1250	*p* value	SMD	ICE, *N* = 1250	No ICE, *N* = 1250	*p* value	SMD
**Age**			.085				.716	
18–54	4933 (32.3)	406 (32.5)		−.004	393 (31.4)	406 (32.5)		−.022
55–60	5629 (36.9)	426 (34.1)		.059	445 (35.6)	426 (34.1)		.032
61+	4712 (30.8)	418 (33.4)		−.055	412 (33.0)	418 (33.4)		−.010
**Female**	4072 (26.7)	325 (26.0)	.635	.015	303 (24.2)	325 (26.0)	.333	−.040
**Elixhauser Score 4**+	8215 (53.8)	652 (52.2)	.281	.033	658 (52.6)	652 (52.2)	.841	.010
**CHADS2VASc Score 2**+	7989 (52.3)	660 (52.8)	.758	−.010	673 (53.8)	660 (52.8)	.630	.021
**Sleep apnea**	6281 (41.1)	448 (35.8)	<.001	.110	448 (35.8)	448 (35.8)	1.000	.000
**AAD use**	9985 (65.4)	644 (51.5)	<.001	.277	633 (50.6)	644 (51.5)	.689	−.018
**Anticoagulant use**	11 749 (76.9)	832 (66.6)	<.001	.220	861 (68.9)	832 (66.6)	.231	.049
**Region**			<.001				.746	
Northeast	2402 (15.7)	375 (30.0)		−.311	384 (30.7)	375 (30.0)		.016
North Central	3405 (22.3)	142 (11.4)		.345	136 (10.9)	142 (11.4)		−.015
South	7305 (47.8)	527 (42.2)		.115	538 (43.0)	527 (42.2)		.018
West	2139 (14.0)	201 (16.1)		−.057	190 (15.2)	201 (16.1)		−.024
Unknown	23 (0.2)	5 (0.4)		−.040	2 (0.2)	5 (0.4)		−.038
**Insurance**			.216				.430	
Basic or comprehensive	527 (3.5)	46 (3.7)		−.012	33 (2.6)	46 (3.7)		−.055
CDHP/HDHP	3119 (20.4)	248 (19.8)		.015	251 (20.1)	248 (19.8)		.006
EPO/HMO	1641 (10.7)	150 (12.0)		−.039	143 (11.4)	150 (12.0)		−.017
POS	1072 (7.0)	104 (8.3)		−.047	114 (9.1)	104 (8.3)		.029
PPO	8643 (56.6)	686 (54.9)		.034	700 (56.0)	686 (54.9)		.023
Unknown	272 (1.8)	16 (1.3)		.045	16 (1.3)	9 (0.7)		−.050
**Year**			<.001				.950	
2016	3913 (25.6)	416 (33.3)		−.163	424 (33.9)	416 (33.3)		.014
2017	3799 (24.9)	318 (25.4)		−.013	318 (25.4)	318 (25.4)		.000
2018	3956 (25.9)	278 (22.2)		.088	281 (22.5)	278 (22.2)		.006
2019	3606 (23.6)	238 (19.0)		.116	227 (18.2)	238 (19.0)		−.022

*Note*: Data are *n* (%).

Abbreviations: AAD, antiarrhythmic drug use; CDHP, consumer‐directed health plan; CHADS2VASc, stands for congestive heart failure, hypertension, age ≥ 75 years, diabetes mellitus, stroke or transient ischemic attack, vascular disease, age 65–74 years, sex category; EPO, exclusive provider organization; HDHP, high deductible health plan; HMO, health maintenance organization; ICE, intracardiac echocardiography; POS, point of service; PPO, preferred provider organization; SMD, standardized mean difference.

Patients in the ICE and non‐ICE groups did not differ in rates of 12‐month inpatient admissions (0–12 months CV‐related inpatient admission rate: 9.4% vs. 10.5%, log‐rank *p* = .394; 0–12 months AF‐related inpatient admission rate: 4.9% vs. 6.0%, log‐rank *p* = .240). However, patients who had CA with ICE had a significantly lower rate of repeat ablation in the 12‐month postprocedure period compared to those who had CA without ICE (7.4% vs. 11.5%, log‐rank *p* = .001). No differences were observed in the rate of electrical cardioversion (10.1% vs. 8.7%, log‐rank *p* = .160). For all healthcare utilization outcomes, no differences were observed among the ICE and non‐ICE patients in the postblanking 4‐ to 12‐month period. Table 4 lists the results for the 0–12 and 4–12 months healthcare utilization outcomes for the ICE and non‐ICE groups.

**Table 4:** Healthcare utilization outcomes among atrial fibrillation (AF) patients 0–12 and 4–12 months after catheter ablation by intracardiac echocardiography (ICE) use

	ICE, *N* = 1250	No ICE, *N* = 1250	Hazard ratio	95% CI	*p* value (log‐rank)	BH adjusted critical value^a^
**0–12 months (no “blanking” period)**						
CV‐related readmission	117 (9.4)	113 (10.5)	0.90	0.70–1.15	.394	.100
AF‐related readmission	61 (4.9)	75 (6.0)	0.82	0.58–1.15	.240	.075
Repeat catheter ablation	93 (7.4)	144 (11.5)	0.64	0.49–0.83	<.001	**.025**
Electrical cardioversion	126 (10.1)	109 (8.7)	1.20	0.93–1.55	.160	.050
**4–12 months (with “blanking” period)**						
CV‐related readmission	61 (4.9%)	64 (5.1)	0.98	0.69–1.39	.905	.200
AF‐related readmission	32 (2.6)	31 (2.5)	1.06	0.65–1.74	.812	.150
Repeat catheter ablation	66 (5.3)	71 (5.7)	0.96	0.69–1.34	.812	.175
Electrical cardioversion	46 (3.7)	43 (3.4)	1.11	0.73–1.68	.624	.125

*Note*: Data are *n* (%).

Abbreviations: BH, Benjamini–Hochberg; CI, confidence interval; CV, cardiovascular.

^a^ Benjamini–Hochberg adjusted critical value after correction for multiple comparisons (total number of tests considered for adjustment = 8; false discovery rate of 20%). After BH adjustment, the largest *p* value which was lower than the BH adjusted critical value was identified. We then designated every *p* value smaller than this *p* value as significant. The adjusted values that are bolded represent significant results.

Results from regression analysis revealed no significant differences in 0–12 months or postblanking 4–12 months CV‐ and AF‐related inpatient admissions and electrical cardioversion among the two study groups. Patients who had CA with ICE had a 36% lower risk of 12‐month repeat ablation compared to those who had CA without ICE (HR 0.64, 95% CI 0.49–0.83). The two groups did not differ significantly in the rate of repeat ablation for the postblanking 4‐ to 12‐month period (Table 4).

## 4 DISCUSSION

In this real‐world study of non‐elderly adult patients with AF, those who underwent an ablation procedure with ICE were 50% less likely to have complication compared to patients who underwent ablation without ICE. Further, patients who underwent CA with ICE had a 36% lower risk of repeat CA in the 12‐month postablation period compared to those who had CA without ICE. Other healthcare utilization outcomes, including inpatient admissions and electrical cardioversion, were not significantly different among the ICE and non‐ICE groups.

Several studies have documented the potential for ICE use to reduce ablation‐related complications by allowing for visualization of the treatment area and early detection of periprocedural complications.^16^, ^17^, ^19^, ^20^, ^21^, ^22^, ^23^, ^24^, ^25^, ^26^ In their analysis of a US hospital discharge database, Isath et al. observed a 28% lower risk of mortality and a 52% lower risk of procedural complications among patients undergoing AF ablation with versus without ICE use,^17^ which aligns well with our results for overall complications.

Our finding that patients undergoing CA with ICE are at significantly lower risk for acute coronary syndrome is also notable. To better understand this difference, we examined the distribution of acute coronary syndrome diagnosis codes among the ICE and non‐ICE cohort. Among patients in the ICE cohort that had experienced acute coronary syndrome (*n* = 10), 40% (4/10) had the ICD‐10‐CM code I21.3 (representing “ST elevation MI [STEMI] of unspecified site”), 30% (3/10) had the code I21.4 (non‐ST elevation MI [NSTEMI]), and the remaining patients were identified based on diagnosis codes I20.0 (Unstable angina), I21.09 (ST elevation MI [STEMI] involving other coronary artery of anterior wall), I21.02 (ST elevation MI [STEMI] involving left anterior descending coronary artery), and I24.0 (acute coronary thrombosis not resulting in MI). Among the 38 patients in the non‐ICE cohort that experienced acute coronary syndrome, almost 45% (17/38) were identified based on diagnosis code I21.4 (non‐ST elevation MI [NSTEMI]), 18% (7/38) were identified based on diagnosis code I21.19 (ST elevation MI [STEMI] involving other coronary artery of inferior wall), 13% (5/38) were identified with code I20.0 (unstable angina), and the remaining were identified based on diagnosis codes I24.8 (other forms of acute ischemic heart disease), I21.29 (ST elevation MI [STEMI] involving other sites), I21.3 (ST elevation MI [STEMI] of unspecified site), I21.09 (ST elevation MI [STEMI] involving other coronary artery of anterior wall), I21.9 (acute MI [unspecified]), and I22.1 (subsequent STEMI inferior wall). Though rare, acute coronary syndrome represents a potential life‐threatening complication that could occur during CA due to injury to the vascular wall. Having tools such as ICE that allow target precision during ablation could alleviate the risk of coronary artery syndrome. The authors hypothesize such events as thromboembolism with resultant coronary thrombosis or alternatively inadvertent aortic puncture leading to thrombus formation and coronary event to be two complications that could be averted with appropriate visualization during CA. The fact that no significant differences were observed on any other specific complications of interest may partially reflect the inadequate power due to the limited sample size.

Differences in repeat ablation rates occurred with the inclusion of the blanking period suggesting potential differential in lesion quality in patients undergoing their CA with versus without ICE. To better understand this variation, we examined the difference in the rate of repeat ablation among the ICE and non‐ICE cohort during the blanking period (0–3 months). A significantly lower rate of repeat ablation was observed during the blanking period among patients in the ICE cohort as compared to those in the non‐ICE cohort (2.2% vs. 5.8%, *p* < .0001). No difference in AF‐related hospitalization rates (2.6% vs. 3.7%, *p* = .1073) or cardioversion (0.8% vs 0.2%, *p* = .0952) was seen during this time among patients in the ICE and non‐ICE cohort. These differences are likely indicative of more accurate visualization of catheter‐tissue contact with ICE use, especially in areas of complex anatomy to ensure optimal lesion creation, which potentially reduces reconduction once the edema subsides within the blanking period. As in the current study, a Medicare fee‐for‐service claims study of AF ablation by Steinberg et al. found that ICE use was associated with a lower risk of repeat ablation (HR 0.59, 95% CI 0.37–0.92).^31^ Though cost was not evaluated as an outcome in the current study, it is likely that improved effectiveness of CA associated with ICE use as observed in our study, may lead to lower healthcare costs for AF management among these patients as compared to those who undergo CA without ICE. Further studies are warranted to explore the potential economic benefits of the use of ICE during ablation procedures.

While ICE use for AF ablation has increased over time, evidence indicates that its adoption has been higher in the US and Canada compared to other countries. In a survey of 60 experts, 53% reported routinely using ICE; however, 87% of members from the United States and Canada indicated using ICE as compared to only 13% of those from other countries.^6^ The reasons for such wide regional variation in adoption of ICE for ablation are not fully understood and may be due to some underlying economic factors. Our study, together with evidence from prior studies, suggests that patients undergoing an ablation procedure with ICE are likely to benefit from a safety and effectiveness perspective. Further, as healthcare dollars are limited, improved outcomes with ICE use are likely to translate into improved cost savings over the long term for patients, providers, and payers.

## 5 LIMITATIONS

This study has several limitations. Since we relied on administrative claims data, which are typically generated for insurance claim adjudication purposes, important procedural details including ablation strategy, use of mapping systems, type of ablation catheter used, and procedure or fluoroscopy time were missing and could not be studied. Another confounding factor that could influence outcomes among patients undergoing ablation is hospital or provider volume. As hospital or provider identifiers are not included in the database studied, we could not control for the effect of volume in our analyses. Further, healthcare utilization was used as a proxy for clinical AF recurrence. Given the database used does not include elderly individuals, we had to restrict the upper age for our sample to 64 years of age. As such, our results may not be generalizable to elderly patients or non‐elderly adult patients with non‐private insurance (government‐sponsored insurance or self‐pay). Given the use of claims data, coding errors, missing data, and reporting bias may also be present and could have influenced study results.

## 6 CONCLUSION

In our analysis of administrative claims data for non‐elderly adult patients with AF in the US, those who had an ablation procedure with ICE had significantly lower risk of having a complication and lower likelihood of needing a repeat ablation as compared to those who had the procedure without ICE. Our results suggest ICE use is associated with the significant clinical benefit of improved safety and effectiveness in AF ablation procedures. Further consideration should be given to the cost impact of these improved outcomes on overall AF management among patients undergoing ablation procedure.

## Supporting information

Supplementary information.

## Data Availability

Restrictions apply to the availability of these data, which were used under license for this study.

## APPENDIX A International Classification of Diseases, 9th and 10th revision (ICD‐9 and ICD‐10) codes and current procedural terminology (CPT) for independent variables, healthcare utilization outcomes, and inclusion/exclusion criteria

**Table:**

Procedure	ICD‐9	ICD‐10	CPT
Intracardiac echocardiography (ICE)		B244YZZ, B244ZZZ, B245YZZ, B245ZZZ, B246YZZ, B246ZZZ, B24DYZZ, B24DZZZ	93662
**Healthcare utilization outcomes/inclusion criteria**			
Atrial fibrillation		I48.0, I48.1x, I48.2x, I48.91	
Catheter ablation		02553ZZ, 02563ZZ, 02573ZZ, 02583ZZ, 025K3ZZ, 025L3ZZ, 025M3ZZ, 025S3ZZ, 025T3ZZ	93651, 93656
Cardiovascular‐related readmission		I00.xx‐I99.xx	
Electrical cardioversion		5A2204Z, 5A12012	92960, 92961
**Exclusion criteria**			
Valvular procedures	35.00, 35.01, 35.02, 35.03, 35.04, 35.05, 35.06, 35.07, 35.08, 35.09, 35.10, 35.11, 35.12, 35.13, 35.14, 35.20, 35.21, 35.22, 35.23, 35.24, 35.25, 35.26, 35.27, 35.28, 35.31, 35.32, 35.33, 35.34, 35.35, 35.39, 35.41, 35.42, 35.50, 35.51, 35.52, 35.53, 35.54, 35.55, 35.60, 35.61, 35.62, 35.63, 35.70, 35.71, 35.72, 35.73, 35.81, 35.82, 35.83, 35.84, 35.91, 35.92, 35.93, 35.94, 35.95, 35.96, 35.97, 35.98, 35.99	021608P, 021608Q, 021608R, 021609P, 021609Q, 021609R, 02160AP, 02160AQ, 02160AR, 02160JP, 02160JQ, 02160JR, 02160KP, 02160KQ, 02160KR, 02160ZP, 02160ZQ, 02160ZR, 021648P, 021648Q, 021648R, 021649P, 021649Q, 021649R, 02164AP, 02164AQ, 02164AR, 02164JP, 02164JQ, 02164JR, 02164KP, 02164KQ, 02164KR, 02164ZP, 02164ZQ, 02164ZR, 021708P, 021708Q, 021708R, 021708S, 021708T, 021708U, 021709P, 021709Q, 021709R, 021709S, 021709T, 021709U, 02170AP, 02170AQ, 02170AR, 02170AS, 02170AT, 02170AU, 02170JP, 02170JQ, 02170JR, 02170JS, 02170JT, 02170JU, 02170KP, 02170KQ, 02170KR, 02170KS, 02170KT, 02170KU, 02170ZP, 02170ZQ, 02170ZR, 02170ZS, 02170ZT, 02170ZU, 021748P, 021748Q, 021748R, 021748S, 021748, 021748U, 021749P, 021749Q, 021749R, 021749S, 021749T, 021749U, 02174AP, 02174AQ, 02174AR, 02174AS, 02174AT, 02174AU, 02174JP, 02174JQ, 02174JR, 02174JS, 02174JT, 02174JU, 02174KP, 02174KQ, 02174KR, 02174KS, 02174KT, 02174KU, 02174ZP, 02174ZQ, 02174ZR, 02174ZS, 02174ZT, 02174ZU, 021K08P, 021K08Q, 021K08R, 021K09P, 021K09Q, 021K09R, 021K0AP, 021K0AQ, 021K0AR, 021K0JP, 021K0JQ, 021K0JR, 021K0KP, 021K0KQ, 021K0KR, 021K0ZP, 021K0ZQ, 021K0ZR, 021K48P, 021K48Q, 021K48R, 021K49P, 021K49Q, 021K49R, 021K4AP, 021K4AQ, 021K4AR, 021K4JP, 021K4JQ, 021K4JR, 021K4KP, 021K4KQ, 021K4KR, 021K4ZP, 021K4ZQ, 021K4ZR, 021L0ZW, 021L4ZW, 021V08S, 021V08T, 021V08U, 021V09S, 021V09T, 021V09U, 021V0AS, 021V0AT, 021V0AU, 021V0JS, 021V0JT, 021V0JU, 021V0KS, 021V0KT, 021V0KU, 021V0ZS, 021V0ZT, 021V0ZU, 021V48S, 021V48T, 021V48U, 021V49S, 021V49T, 021V49U, 021V4AS, 021V4AT, 021V4AU, 021V4JS, 021V4JT, 021V4JU, 021V4KS, 021V4KT, 021V4KU, 021V4ZS, 021V4ZT, 021V4ZU, 024F07J, 024F08J, 024F0JJ, 024F0KJ, 024G072, 024G082, 024G0J2, 024G0K2, 024J072, 024J082, 024J0J2, 024J0K2, 02590ZZ, 02593ZZ, 02594ZZ, 025D0ZZ, 025D3ZZ, 025D4ZZ, 027F04Z, 027F0DZ, 027F0ZZ, 027F34Z, 027F3DZ, 027F3ZZ, 027F44Z, 027F4DZ, 027F4ZZ, 027G04Z, 027G0DZ, 027G0ZZ, 027G34Z, 027G3DZ, 027G3ZZ, 027G44Z, 027G4DZ, 027G4ZZ, 027H04Z, 027H0DZ, 027H0ZZ, 027H34Z, 027H3DZ, 027H3ZZ, 027H44Z, 027H4DZ, 027H4ZZ, 027J04Z, 027J0DZ, 027J0ZZ, 027J34Z, 027J3DZ, 027J3ZZ, 027J44Z, 027J4DZ, 027J4ZZ, 02890ZZ, 02893ZZ, 02894ZZ, 028D0ZZ, 028D3ZZ, 028D4ZZ, 02B50ZZ, 02B53ZZ, 02B54ZZ, 02B90ZX, 02B90ZZ, 02B93ZX, 02B93ZZ, 02B94ZX, 02B94ZZ, 02BD0ZZ, 02BD3ZZ, 02BD4ZZ, 02BK0ZX, 02BK0ZZ, 02BK3ZX, 02BK3ZZ, 02BK4ZX, 02BK4ZZ, 02BL0ZX, 02BL0ZZ, 02BL3ZX, 02BL3ZZ, 02BL4ZX, 02BL4ZZ, 02C50ZZ, 02C53ZZ, 02C54ZZ, 02C90ZZ, 02C93ZZ, 02C94ZZ, 02CD0ZZ, 02CD3ZZ, 02CD4ZZ, 02CF0ZZ, 02CF3ZZ, 02CF4ZZ, 02CG0ZZ, 02CG3ZZ, 02CG4ZZ, 02CH0ZZ, 02CH3ZZ, 02CH4ZZ, 02CJ0ZZ, 02CJ3ZZ, 02CJ4ZZ, 02CM0ZZ, 02CM3ZZ, 02CM4ZZ, 02LH0CZ, 02LH0DZ, 02LH0ZZ, 02LH3CZ, 02LH3DZ, 02LH3ZZ, 02LH4CZ, 02LH4DZ, 02LH4ZZ, 02LR0ZT, 02LS0ZZ, 02LT0ZZ, 02N50ZZ, 02N53ZZ, 02N54ZZ, 02N94ZZ, 02ND0ZZ, 02ND3ZZ, 02ND4ZZ, 02NF0ZZ, 02NF3ZZ, 02NF4ZZ, 02NG0ZZ, 02NG3ZZ, 02NG4ZZ, 02NH0ZZ, 02NH3ZZ, 02NH4ZZ, 02NJ0ZZ, 02NJ3ZZ, 02NJ4ZZ, 02NK0ZZ, 02NK3ZZ, 02NK4ZZ, 02NL0ZZ, 02NL3ZZ, 02NL4ZZ, 02NM0ZZ, 02NM3ZZ, 02NM4ZZ, 02Q50ZZ, 02Q53ZZ, 02Q54ZZ, 02Q90ZZ, 02Q94ZZ, 02QA0ZZ, 02QA3ZZ, 02QA4ZZ, 02QB0ZZ, 02QB3ZZ, 02QB4ZZ, 02QC0ZZ, 02QC3ZZ, 02QC4ZZ, 02QD0ZZ, 02QD3ZZ, 02QD4ZZ, 02QF0ZJ, 02QF0ZZ, 02QF3ZJ, 02QF3ZZ, 02QF4ZJ, 02QF4ZZ, 02QG0ZE, 02QG0ZZ, 02QG3ZE, 02QG3ZZ, 02QG4ZE, 02QG4ZZ, 02QH0ZZ, 02QH3ZZ, 02QH4ZZ, 02QJ0ZG, 02QJ0ZZ, 02QJ3ZG, 02QJ3ZZ, 02QJ4ZG, 02QJ4ZZ, 02QM0ZZ, 02QM3ZZ, 02QM4ZZ, 02R50JZ, 02R907Z, 02R908Z, 02R947Z, 02R948Z, 02R94JZ, 02R94KZ, 02RD07Z, 02RD08Z, 02RD0JZ, 02RD0KZ, 02RD47Z, 02RD48Z, 02RD4JZ, 02RD4KZ, 02RF07Z, 02RF08Z, 02RF0JZ, 02RF0KZ, 02RF37H, 02RF37Z, 02RF38H, 02RF38Z, 02RF3JH, 02RF3JZ, 02RF3KH, 02RF3KZ, 02RF47Z, 02RF48Z, 02RF4JZ, 02RF4KZ, 02RG07Z, 02RG08Z, 02RG0JZ, 02RG0KZ, 02RG37H, 02RG37Z, 02RG38H, 02RG38Z, 02RG3JH, 02RG3JZ, 02RG3KH, 02RG3KZ, 02RG47Z, 02RG48Z, 02RG4JZ, 02RG4KZ, 02RH07Z, 02RH08Z, 02RH0JZ, 02RH0KZ, 02RH37H, 02RH37Z, 02RH38H, 02RH38Z, 02RH3JH, 02RH3JZ, 02RH3KH, 02RH3KZ, 02RH47Z, 02RH48Z, 02RH4JZ, 02RH4KZ, 02RJ07Z, 02RJ08Z, 02RJ0JZ, 02RJ0KZ, 02RJ37H, 02RJ37Z, 02RJ38H, 02RJ38Z, 02RJ3JH, 02RJ3JZ, 02RJ3KH, 02RJ3KZ, 02RJ47Z, 02RJ48Z, 02RJ4JZ, 02RJ4KZ, 02RK07Z, 02RK0KZ, 02RK47Z, 02RK4KZ, 02RL07Z, 02RL0KZ, 02RL47Z, 02RL4KZ, 02RM07Z, 02RM0JZ, 02RM0KZ, 02RM47Z, 02RM4JZ, 02RM4KZ, 02RP0JZ, 02RQ07Z, 02RQ0JZ, 02RR07Z, 02RR0JZ, 02S00ZZ, 02S10ZZ, 02SP0ZZ, 02SW0ZZ, 02SX0ZZ, 02T50ZZ, 02T53ZZ, 02T54ZZ, 02T90ZZ, 02T93ZZ, 02T94ZZ, 02TD0ZZ, 02TD3ZZ, 02TD4ZZ, 02TH0ZZ, 02TH3ZZ, 02TH4ZZ, 02TM0ZZ, 02TM3ZZ, 02TM4ZZ, 02U507Z, 02U508Z, 02U50JZ, 02U50KZ, 02U537Z, 02U538Z, 02U53JZ, 02U53KZ, 02U547Z, 02U548Z, 02U54JZ, 02U54KZ, 02U607Z, 02U608Z, 02U60KZ, 02U707Z, 02U708Z, 02U70JZ, 02U70KZ, 02U737Z, 02U738Z, 02U73KZ, 02U747Z, 02U748Z, 02U74KZ, 02U907Z, 02U908Z, 02U90JZ, 02U90KZ, 02U937Z, 02U938Z, 02U93JZ, 02U93KZ, 02U947Z, 02U948Z, 02U94JZ, 02U94KZ, 02UD07Z, 02UD08Z, 02UD0JZ, 02UD0KZ, 02UD37Z, 02UD38Z, 02UD3JZ, 02UD3KZ, 02UD47Z, 02UD48Z, 02UD4JZ, 02UD4KZ, 02UF07J, 02UF07Z, 02UF08J, 02UF08Z, 02UF0JJ, 02UF0JZ, 02UF0KJ, 02UF0KZ, 02UF37J, 02UF37Z, 02UF38J, 02UF38Z, 02UF3JJ, 02UF3JZ, 02UF3KJ, 02UF3KZ, 02UF47J, 02UF47Z, 02UF48J, 02UF48Z, 02UF4JJ, 02UF4JZ, 02UF4KJ, 02UF4KZ, 02UG07E, 02UG07Z, 02UG08E, 02UG08Z, 02UG0JE, 02UG0JZ, 02UG0KE, 02UG0KZ, 02UG37E, 02UG37Z, 02UG38E, 02UG38Z, 02UG3JE, 02UG3JZ, 02UG3KE, 02UG3KZ, 02UG47E, 02UG47Z, 02UG48E, 02UG48Z, 02UG4JE, 02UG4JZ, 02UG4KE, 02UG4KZ, 02UH07Z, 02UH08Z, 02UH0JZ, 02UH0KZ, 02UH37Z, 02UH38Z, 02UH3JZ, 02UH3KZ, 02UH47Z, 02UH48Z, 02UH4JZ, 02UH4KZ, 02UJ07G, 02UJ07Z, 02UJ08G, 02UJ08Z, 02UJ0JG, 02UJ0JZ, 02UJ0KG, 02UJ0KZ, 02UJ37G, 02UJ37Z, 02UJ38G, 02UJ38Z, 02UJ3JG, 02UJ3JZ, 02UJ3KG, 02UJ3KZ, 02UJ47G, 02UJ47Z, 02UJ48G, 02UJ48Z, 02UJ4JG, 02UJ4JZ, 02UJ4KG, 02UJ4KZ, 02UK0KZ, 02UK3KZ, 02UK4KZ, 02UL0KZ, 02UL3KZ, 02UL4KZ, 02UM07Z, 02UM0JZ, 02UM0KZ, 02UM37Z, 02UM38Z, 02UM3JZ, 02UM3KZ, 02UM47Z, 02UM48Z, 02UM4JZ, 02UM4KZ, 02UP07Z, 02UP0JZ, 02UP0KZ, 02UP37Z, 02UP3JZ, 02UP3KZ, 02UP47Z, 02UP4JZ, 02UP4KZ, 02UQ07Z, 02UQ0JZ, 02UQ0KZ, 02UQ37Z, 02UQ3JZ, 02UQ3KZ, 02UQ47Z, 02UQ4JZ, 02UQ4KZ, 02UW07Z, 02VG0ZZ, 02VG3ZZ, 02VG4ZZ, 02VR0ZT, 02W50JZ, 02W54JZ, 02WF07Z, 02WF08Z, 02WF0JZ, 02WF0KZ, 02WF37Z, 02WF38Z, 02WF3JZ, 02WF3KZ, 02WF47Z, 02WF48Z, 02WF4JZ, 02WF4KZ, 02WG07Z, 02WG08Z, 02WG0JZ, 02WG0KZ, 02WG37Z, 02WG38Z, 02WG3JZ, 02WG3KZ, 02WG47Z, 02WG48Z, 02WG4JZ, 02WG4KZ, 02WH07Z, 02WH08Z, 02WH0JZ, 02WH0KZ, 02WH37Z, 02WH38Z, 02WH3JZ, 02WH3KZ, 02WH47Z, 02WH48Z, 02WH4JZ, 02WH4KZ, 02WJ07Z, 02WJ08Z, 02WJ0JZ, 02WJ0KZ, 02WJ37Z, 02WJ38Z, 02WJ3JZ, 02WJ3KZ, 02WJ47Z, 02WJ48Z, 02WJ4JZ, 02WJ4KZ, 02WM0JZ, 02WM4JZ, X2RF032, X2RF332, X2RF432	3361, 33362, 33363, 33364, 33365, 33366, 33367, 33368, 33369, 33400, 33401, 33403, 33405, 33406, 33410, 33411, 33412, 33413, 33414, 33415, 33416, 33417, 33418, 33419, 33420, 33422, 33425, 33426, 33427, 33430, 33460, 33463, 33464, 33465, 33468, 33470, 33471, 33474, 33475, 33476, 33477, 33478, 33496, 33600, 33602, 33390, 33391, 34501
Atrioventricular node ablation	N/A	N/A	93650
Left atrial appendage occlusion	37.36, 37.90	02570ZK, 02573ZK, 02574ZK, 02B70ZK, 02B73ZK, 02B74ZK, 02H73DZ, 02H74DZ, 02L70CK, 02L70DK, 02L70ZK, 02L73CK, 02L73DK, 02L73ZK, 02L74CK, 02L74DK, 02L74ZK	N/A
Surgical ablation	37.33, 37.37	02550ZZ, 02560ZZ, 02570ZZ, 02580ZZ, 02590ZZ, 025F0ZZ, 025G0ZZ, 025H0ZZ, 025J0ZZ, 025K0ZZ, 025L0ZZ, 025M0ZZ, 02B50ZZ, 02B60ZZ, 02B70ZZ, 02B80ZZ, 02B90ZZ, 02BF0ZZ, 02BG0ZZ, 02BH0ZZ, 02BJ0ZZ, 02BK0ZZ, 02BL0ZZ, 02BM0ZZ, 02T80ZZ, 02554ZZ, 02564ZZ, 02574ZZ, 02584ZZ, 02594ZZ, 025F4ZZ, 025G4ZZ, 025H4ZZ, 025J4ZZ, 025K4ZZ, 025L4ZZ, 025M4ZZ, 02B54ZZ, 02B64ZZ, 02B74ZZ, 02B84ZZ, 02B94ZZ, 02BF4ZZ, 02BG4ZZ, 02BH4ZZ, 02BJ4ZZ, 02BK4ZZ, 02BL4ZZ, 02BM4ZZ, 02T84ZZ	33250, 33251, 33252, 33253, 33254, 33255, 33256, 33257, 33258, 33259, 33261, 33265, 33266

Abbreviations: CPT, current procedural terminology; ICD‐9, International Classification of Diseases, 9th revision; ICD‐10, International Classification of Diseases, 10th revision.

## APPENDIX B International Classification of Diseases, 9th and 10th revision (ICD‐9 and ICD‐10) codes, current procedural terminology (CPT) codes, and Healthcare Common Procedure Coding System (HCPCS) codes for included complications

Laryngospasm:

**Table:**

Code type	Code	Code description
ICD‐10‐CM	J38.5	Laryngeal spasm

Mandibular Joint Dislocation:

**Table:**

Code type	Code	Code description
ICD‐10‐CM	S03.00XA	Dislocation of jaw, unspecified – first encounter
ICD‐10‐CM	S03.01XA	Dislocation of jaw, right side – first encounter
ICD‐10‐CM	S03.02XA	Dislocation of jaw, left side – first encounter
ICD‐10‐CM	S03.03XA	Dislocation of jaw, bilateral – first encounter

Acute Coronary Syndrome:

**Table:**

Code type	Code	Code description
ICD‐10‐CM	I20.0	Unstable angina
ICD‐10‐CM	I21.0	ST elevation MI (STEMI) of anterior wall
ICD‐10‐CM	I21.01	ST elevation MI (STEMI) involving left main coronary artery
ICD‐10‐CM	I21.02	ST elevation MI (STEMI) involving left anterior descending coronary artery
ICD‐10‐CM	I21.09	ST elevation MI (STEMI) involving other coronary artery of anterior wall
ICD‐10‐CM	I21.1	ST elevation MI (STEMI) of inferior wall
ICD‐10‐CM	I21.11	ST elevation MI (STEMI) involving right coronary artery
ICD‐10‐CM	I21.19	ST elevation MI (STEMI) involving other coronary artery of inferior wall
ICD‐10‐CM	I21.2	ST elevation MI (STEMI) of other sites
ICD‐10‐CM	I21.21	ST elevation MI (STEMI) involving left circumflex coronary artery
ICD‐10‐CM	I21.29	ST elevation MI (STEMI) involving other sites
ICD‐10‐CM	I21.3	ST elevation MI (STEMI) of unspecified site
ICD‐10‐CM	I21.4	Non‐ST elevation MI (NSTEMI)
ICD‐10‐CM	I21.9	Acute MI (unspecified)
ICD‐10‐CM	I22.0	Subsequent STEMI anterior wall
ICD‐10‐CM	I22.1	Subsequent STEMI inferior wall
ICD‐10‐CM	I22.2	Subsequent NSTEMI
ICD‐10‐CM	I22.8	Subsequent STEMI other sites
ICD‐10‐CM	I22.9	Subsequent STEMI unspecified site
ICD‐10‐CM	I24.0	Acute coronary thrombosis not resulting in MI
ICD‐10‐CM	I24.8	Other forms of acute ischemic heart disease

Ischemic Stroke:

**Table:**

Code type	Code	Code description
ICD‐10‐CM	I63.00	Cerebral infarction due to thrombosis of unspecified precerebral artery
ICD‐10‐CM	I63.011	Cerebral infarction due to thrombosis of right vertebral artery
ICD‐10‐CM	I63.012	Cerebral infarction due to thrombosis of left vertebral artery
ICD‐10‐CM	I63.013	Cerebral infarction due to thrombosis of bilateral vertebral arteries
ICD‐10‐CM	I63.019	Cerebral infarction due to thrombosis of unspecified vertebral artery
ICD‐10‐CM	I63.02	Cerebral infarction due to thrombosis of basilar artery
ICD‐10‐CM	I63.031	Cerebral infarction due to thrombosis of right carotid artery
ICD‐10‐CM	I63.032	Cerebral infarction due to thrombosis of left carotid artery
ICD‐10‐CM	I63.033	Cerebral infarction due to thrombosis of bilateral carotid arteries
ICD‐10‐CM	I63.039	Cerebral infarction due to thrombosis of unspecified carotid artery
ICD‐10‐CM	I63.09	Cerebral infarction due to thrombosis of other precerebral artery
ICD‐10‐CM	I63.10	Cerebral infarction due to embolism of unspecified precerebral artery
ICD‐10‐CM	I63.111	Cerebral infarction due to embolism of right vertebral artery
ICD‐10‐CM	I63.112	Cerebral infarction due to embolism of left vertebral artery
ICD‐10‐CM	I63.113	Cerebral infarction due to embolism of bilateral vertebral arteries
ICD‐10‐CM	I63.119	Cerebral infarction due to embolism of unspecified vertebral artery
ICD‐10‐CM	I63.12	Cerebral infarction due to embolism of basilar artery
ICD‐10‐CM	I63.131	Cerebral infarction due to embolism of right carotid artery
ICD‐10‐CM	I63.132	Cerebral infarction due to embolism of left carotid artery
ICD‐10‐CM	I63.133	Cerebral infarction due to embolism of bilateral carotid arteries
ICD‐10‐CM	I63.139	Cerebral infarction due to embolism of unspecified carotid artery
ICD‐10‐CM	I63.19	Cerebral infarction due to embolism of other precerebral artery
ICD‐10‐CM	I63.20	Cerebral infarction due to unspecified occlusion or stenosis of unspecified precerebral arteries
ICD‐10‐CM	I63.211	Cerebral infarction due to unspecified occlusion or stenosis of right vertebral artery
ICD‐10‐CM	I63.212	Cerebral infarction due to unspecified occlusion or stenosis of left vertebral artery
ICD‐10‐CM	I63.213	Cerebral infarction due to unspecified occlusion or stenosis of bilateral vertebral arteries
ICD‐10‐CM	I63.219	Cerebral infarction due to unspecified occlusion or stenosis of unspecified vertebral arteries
ICD‐10‐CM	I63.22	Cerebral infarction due to unspecified occlusion or stenosis of basilar artery
ICD‐10‐CM	I63.231	Cerebral infarction due to unspecified occlusion or stenosis of right carotid arteries
ICD‐10‐CM	I63.232	Cerebral infarction due to unspecified occlusion or stenosis of left carotid arteries
ICD‐10‐CM	I63.233	Cerebral infarction due to unspecified occlusion or stenosis of bilateral carotid arteries
ICD‐10‐CM	I63.239	Cerebral infarction due to unspecified occlusion or stenosis of unspecified carotid arteries
ICD‐10‐CM	I63.29	Cerebral infarction due to unspecified occlusion or stenosis of other precerebral arteries
ICD‐10‐CM	I63.30	Cerebral infarction due to thrombosis of unspecified cerebral artery
ICD‐10‐CM	I63.311	Cerebral infarction due to thrombosis of right middle cerebral artery
ICD‐10‐CM	I63.312	Cerebral infarction due to thrombosis of left middle cerebral artery
ICD‐10‐CM	I63.313	Cerebral infarction due to thrombosis of bilateral middle cerebral arteries
ICD‐10‐CM	I63.319	Cerebral infarction due to thrombosis of unspecified middle cerebral artery
ICD‐10‐CM	I63.321	Cerebral infarction due to thrombosis of right anterior cerebral artery
ICD‐10‐CM	I63.322	Cerebral infarction due to thrombosis of left anterior cerebral artery
ICD‐10‐CM	I63.323	Cerebral infarction due to thrombosis of bilateral anterior cerebral arteries
ICD‐10‐CM	I63.329	Cerebral infarction due to thrombosis of unspecified anterior cerebral artery
ICD‐10‐CM	I63.331	Cerebral infarction due to thrombosis of right posterior cerebral artery
ICD‐10‐CM	I63.332	Cerebral infarction due to thrombosis of left posterior cerebral artery
ICD‐10‐CM	I63.333	Cerebral infarction to thrombosis of bilateral posterior cerebral arteries
ICD‐10‐CM	I63.339	Cerebral infarction due to thrombosis of unspecified posterior cerebral artery
ICD‐10‐CM	I63.341	Cerebral infarction due to thrombosis of right cerebellar artery
ICD‐10‐CM	I63.342	Cerebral infarction due to thrombosis of left cerebellar artery
ICD‐10‐CM	I63.343	Cerebral infarction to thrombosis of bilateral cerebellar arteries
ICD‐10‐CM	I63.349	Cerebral infarction due to thrombosis of unspecified cerebellar artery
ICD‐10‐CM	I63.39	Cerebral infarction due to thrombosis of other cerebral artery
ICD‐10‐CM	I63.40	Cerebral infarction due to embolism of unspecified cerebral artery
ICD‐10‐CM	I63.411	Cerebral infarction due to embolism of right middle cerebral artery
ICD‐10‐CM	I63.412	Cerebral infarction due to embolism of left middle cerebral artery
ICD‐10‐CM	I63.413	Cerebral infarction due to embolism of bilateral middle cerebral arteries
ICD‐10‐CM	I63.419	Cerebral infarction due to embolism of unspecified middle cerebral artery
ICD‐10‐CM	I63.421	Cerebral infarction due to embolism of right anterior cerebral artery
ICD‐10‐CM	I63.422	Cerebral infarction due to embolism of left anterior cerebral artery
ICD‐10‐CM	I63.423	Cerebral infarction due to embolism of bilateral anterior cerebral arteries
ICD‐10‐CM	I63.429	Cerebral infarction due to embolism of unspecified anterior cerebral artery
ICD‐10‐CM	I63.431	Cerebral infarction due to embolism of right posterior cerebral artery
ICD‐10‐CM	I63.432	Cerebral infarction due to embolism of left posterior cerebral artery
ICD‐10‐CM	I63.433	Cerebral infarction due to embolism of bilateral posterior cerebral arteries
ICD‐10‐CM	I63.439	Cerebral infarction due to embolism of unspecified posterior cerebral artery
ICD‐10‐CM	I63.441	Cerebral infarction due to embolism of right cerebellar artery
ICD‐10‐CM	I63.442	Cerebral infarction due to embolism of left cerebellar artery
ICD‐10‐CM	I63.443	Cerebral infarction due to embolism of bilateral cerebellar arteries
ICD‐10‐CM	I63.449	Cerebral infarction due to embolism of unspecified cerebellar artery
ICD‐10‐CM	I63.49	Cerebral infarction due to embolism of other cerebral artery
ICD‐10‐CM	I63.50	Cerebral infarction due to unspecified occlusion or stenosis of unspecified cerebral artery
ICD‐10‐CM	I63.511	Cerebral infarction due to unspecified occlusion or stenosis of right middle cerebral artery
ICD‐10‐CM	I63.512	Cerebral infarction due to unspecified occlusion or stenosis of left middle cerebral artery
ICD‐10‐CM	I63.513	Cerebral infarction due to unspecified occlusion or stenosis of bilateral middle cerebral arteries
ICD‐10‐CM	I63.519	Cerebral infarction due to unspecified occlusion or stenosis of unspecified middle cerebral artery
ICD‐10‐CM	I63.521	Cerebral infarction due to unspecified occlusion or stenosis of right anterior cerebral artery
ICD‐10‐CM	I63.522	Cerebral infarction due to unspecified occlusion or stenosis of left anterior cerebral artery
ICD‐10‐CM	I63.523	Cerebral infarction due to unspecified occlusion or stenosis of bilateral anterior cerebral arteries
ICD‐10‐CM	I63.529	Cerebral infarction due to unspecified occlusion or stenosis of unspecified anterior cerebral artery
ICD‐10‐CM	I63.531	Cerebral infarction due to unspecified occlusion or stenosis of right posterior cerebral artery
ICD‐10‐CM	I63.532	Cerebral infarction due to unspecified occlusion or stenosis of left posterior cerebral artery
ICD‐10‐CM	I63.533	Cerebral infarction due to unspecified occlusion or stenosis of bilateral posterior cerebral arteries
ICD‐10‐CM	I63.539	Cerebral infarction due to unspecified occlusion or stenosis of unspecified posterior cerebral artery
ICD‐10‐CM	I63.541	Cerebral infarction due to unspecified occlusion or stenosis of right cerebellar artery
ICD‐10‐CM	I63.542	Cerebral infarction due to unspecified occlusion or stenosis of left cerebellar artery
ICD‐10‐CM	I63.543	Cerebral infarction due to unspecified occlusion or stenosis of bilateral cerebellar arteries
ICD‐10‐CM	I63.549	Cerebral infarction due to unspecified occlusion or stenosis of unspecified cerebellar artery
ICD‐10‐CM	I63.59	Cerebral infarction due to unspecified occlusion or stenosis of other cerebral artery
ICD‐10‐CM	I63.6	Cerebral infarction due to cerebral venous thrombosis, nonpyogenic
ICD‐10‐CM	I63.8	Other cerebral infarction
ICD‐10‐CM	I63.81	Other cerebral infarction due to occlusion or stenosis of small artery
ICD‐10‐CM	I63.89	Other cerebral infarction
ICD‐10‐CM	I63.9	Cerebral infarction, unspecified
ICD‐10‐CM	I97.810	Intraoperative cerebrovascular infarction during cardiac surgery
ICD‐10‐CM	I97.811	Intraoperative cerebrovascular infarction during other surgery
ICD‐10‐CM	I97.820	Postprocedural cerebrovascular infarction following cardiac surgery
ICD‐10‐CM	I97.821	Postprocedural cerebrovascular infarction following other surgery

Hemorrhagic Stroke:

**Table:**

Code type	Code	Code description
ICD‐10‐CM	I60.00	Nontraumatic subarachnoid hemorrhage from unspecified carotid siphon and bifurcation
ICD‐10‐CM	I60.01	Nontraumatic subarachnoid hemorrhage from right carotid siphon and bifurcation
ICD‐10‐CM	I60.02	Nontraumatic subarachnoid hemorrhage from left carotid siphon and bifurcation
ICD‐10‐CM	I60.10	Nontraumatic subarachnoid hemorrhage from unspecified middle cerebral artery
ICD‐10‐CM	I60.11	Nontraumatic subarachnoid hemorrhage from right middle cerebral artery
ICD‐10‐CM	I60.12	Nontraumatic subarachnoid hemorrhage from left middle cerebral artery
ICD‐10‐CM	I60.2	Nontraumatic subarachnoid hemorrhage from anterior communicating artery
ICD‐10‐CM	I60.30	Nontraumatic subarachnoid hemorrhage from unspecified posterior communicating artery
ICD‐10‐CM	I60.31	Nontraumatic subarachnoid hemorrhage from right posterior communicating artery
ICD‐10‐CM	I60.32	Nontraumatic subarachnoid hemorrhage from left posterior communicating artery
ICD‐10‐CM	I60.4	Nontraumatic subarachnoid hemorrhage from basilar artery
ICD‐10‐CM	I60.50	Nontraumatic subarachnoid hemorrhage from unspecified vertebral artery
ICD‐10‐CM	I60.51	Nontraumatic subarachnoid hemorrhage from right vertebral artery
ICD‐10‐CM	I60.52	Nontraumatic subarachnoid hemorrhage from left vertebral artery
ICD‐10‐CM	I60.6	Nontraumatic subarachnoid hemorrhage from other intracranial arteries
ICD‐10‐CM	I60.7	Nontraumatic subarachnoid hemorrhage from unspecified intracranial artery
ICD‐10‐CM	I60.8	Other nontraumatic subarachnoid hemorrhage
ICD‐10‐CM	I60.9	Nontraumatic subarachnoid hemorrhage, unspecified
ICD‐10‐CM	I61.0	Nontraumatic intracerebral hemorrhage in hemisphere, subcortical
ICD‐10‐CM	I61.1	Nontraumatic intracerebral hemorrhage in hemisphere, cortical
ICD‐10‐CM	I61.2	Nontraumatic intracerebral hemorrhage in hemisphere, unspecified
ICD‐10‐CM	I61.3	Nontraumatic intracerebral hemorrhage in brain stem
ICD‐10‐CM	I61.4	Nontraumatic intracerebral hemorrhage in cerebellum
ICD‐10‐CM	I61.5	Nontraumatic intracerebral hemorrhage, intraventricular
ICD‐10‐CM	I61.6	Nontraumatic intracerebral hemorrhage, multiple localized
ICD‐10‐CM	I61.8	Other nontraumatic intracerebral hemorrhage
ICD‐10‐CM	I61.9	Nontraumatic intracerebral hemorrhage, unspecified

Thromboembolism:

**Table:**

Code type	Code	Code description
ICD‐10‐CM	I26.01	Septic pulmonary embolism with acute cor pulmonale
ICD‐10‐CM	I26.02	Saddle embolus of pulmonary artery with acute cor pulmonale
ICD‐10‐CM	I26.09	Other pulmonary embolism with acute cor pulmonale
ICD‐10‐CM	I26.90	Septic pulmonary embolism without acute cor pulmonale
ICD‐10‐CM	I26.92	Saddle embolus of pulmonary artery without acute cor pulmonale
ICD‐10‐CM	I26.93	Single subsegmental pulmonary embolism without acute core pulmonale
ICD‐10‐CM	I26.94	Multiple subsegmental pulmonary emboli without acute cor pulmonale
ICD‐10‐CM	I26.99	Other pulmonary embolism without acute cor pulmonale
ICD‐10‐CM	I82.210	Acute embolism and thrombosis of superior vena cava
ICD‐10‐CM	I82.220	Acute embolism and thrombosis of inferior vena cava
ICD‐10‐CM	I82.290	Acute embolism and thrombosis of other thoracic veins
ICD‐10‐CM	I82.401	Acute embolism and thrombosis of unspecified deep veins of right lower extremity
ICD‐10‐CM	I82.402	Acute embolism and thrombosis of unspecified deep veins of left lower extremity
ICD‐10‐CM	I82.403	Acute embolism and thrombosis of unspecified deep veins of lower extremity, bilateral
ICD‐10‐CM	I82.409	Acute embolism and thrombosis of unspecified deep veins of unspecified lower extremity
ICD‐10‐CM	I82.411	Acute embolism and thrombosis of right femoral vein
ICD‐10‐CM	I82.412	Acute embolism and thrombosis of left femoral vein
ICD‐10‐CM	I82.413	Acute embolism and thrombosis of femoral vein, bilateral
ICD‐10‐CM	I82.419	Acute embolism and thrombosis of unspecified femoral vein
ICD‐10‐CM	I82.421	Acute embolism and thrombosis of right iliac vein
ICD‐10‐CM	I82.422	Acute embolism and thrombosis of left iliac vein
ICD‐10‐CM	I82.423	Acute embolism and thrombosis of iliac vein, bilateral
ICD‐10‐CM	I82.429	Acute embolism and thrombosis of unspecified iliac vein
ICD‐10‐CM	I82.431	Acute embolism and thrombosis of right popliteal vein
ICD‐10‐CM	I82.432	Acute embolism and thrombosis of left popliteal vein
ICD‐10‐CM	I82.433	Acute embolism and thrombosis of popliteal vein, lateral
ICD‐10‐CM	I82.439	Acute embolism and thrombosis of unspecified popliteal vein
ICD‐10‐CM	I82.441	Acute embolism and thrombosis of right tibial vein
ICD‐10‐CM	I82.442	Acute embolism and thrombosis of left tibial vein
ICD‐10‐CM	I82.443	Acute embolism and thrombosis of tibial vein, bilateral
ICD‐10‐CM	I82.449	Acute embolism and thrombosis of unspecified tibial vein
ICD‐10‐CM	I82.451	Acute embolism and thrombosis of right peroneal vein
ICD‐10‐CM	I82.452	Acute embolism and thrombosis of left peroneal vein
ICD‐10‐CM	I82.453	Acute embolism and thrombosis of peroneal vein, bilateral
ICD‐10‐CM	I82.459	Acute embolism and thrombosis of unspecified peroneal vein
ICD‐10‐CM	I82.461	Acute embolism and thrombosis of right calf muscular vein
ICD‐10‐CM	I82.462	Acute embolism and thrombosis of left calf muscular vein
ICD‐10‐CM	I82.463	Acute embolism and thrombosis of calf muscular vein, bilateral
ICD‐10‐CM	I82.469	Acute embolism and thrombosis of unspecified calf muscular vein
ICD‐10‐CM	I82.491	Acute embolism and thrombosis of other specified deep vein of right lower extremity
ICD‐10‐CM	I82.492	Acute embolism and thrombosis of other specified deep vein of left lower extremity
ICD‐10‐CM	I82.493	Acute embolism and thrombosis of other specified deep vein of lower extremity, bilateral
ICD‐10‐CM	I82.499	Acute embolism and thrombosis of other specified deep vein of unspecified lower extremity
ICD‐10‐CM	I82.4Y1	Acute embolism and thrombosis of unspecified deep veins of right proximal lower extremity
ICD‐10‐CM	I82.4Y2	Acute embolism and thrombosis of unspecified deep veins of left proximal lower extremity
ICD‐10‐CM	I82.4Y3	Acute embolism and thrombosis of unspecified deep veins of proximal lower extremity, bilateral
ICD‐10‐CM	I82.4Y9	Acute embolism and thrombosis of unspecified deep veins of unspecified proximal lower extremity
ICD‐10‐CM	I82.4Z1	Acute embolism and thrombosis of unspecified deep veins of right distal lower extremity
ICD‐10‐CM	I82.4Z2	Acute embolism and thrombosis of unspecified deep veins of left distal lower extremity
ICD‐10‐CM	I82.4Z3	Acute embolism and thrombosis of unspecified deep veins of distal lower extremity, bilateral
ICD‐10‐CM	I82.4Z9	Acute embolism and thrombosis of unspecified deep veins of unspecified distal lower extremity
ICD‐10‐CM	I82.601	Acute embolism and thrombosis of unspecified veins of right upper extremity
ICD‐10‐CM	I82.602	Acute embolism and thrombosis of unspecified veins of left upper extremity
ICD‐10‐CM	I82.603	Acute embolism and thrombosis of unspecified veins of upper extremity, bilateral
ICD‐10‐CM	I82.609	Acute embolism and thrombosis of unspecified veins of unspecified upper extremity
ICD‐10‐CM	I82.611	Acute embolism and thrombosis of superficial veins of right upper extremity
ICD‐10‐CM	I82.612	Acute embolism and thrombosis of superficial veins of left upper extremity
ICD‐10‐CM	I82.613	Acute embolism and thrombosis of superficial veins of upper extremity, bilateral
ICD‐10‐CM	I82.619	Acute embolism and thrombosis of superficial veins of unspecified upper extremity
ICD‐10‐CM	I82.621	Acute embolism and thrombosis of deep veins of right upper extremity
ICD‐10‐CM	I82.622	Acute embolism and thrombosis of deep veins of left upper extremity
ICD‐10‐CM	I82.623	Acute embolism and thrombosis of deep veins of upper extremity, bilateral
ICD‐10‐CM	I82.629	Acute embolism and thrombosis of deep veins of unspecified upper extremity
ICD‐10‐CM	I82.890	Acute embolism and thrombosis of other specified veins
ICD‐10‐CM	I82.90	Acute embolism and thrombosis of unspecified vein
ICD‐10‐CM	I82.A11	Acute embolism and thrombosis of right axillary vein
ICD‐10‐CM	I82.A12	Acute embolism and thrombosis of left axillary vein
ICD‐10‐CM	I82.A13	Acute embolism and thrombosis of axillary vein, bilateral
ICD‐10‐CM	I82.A19	Acute embolism and thrombosis of unspecified axillary vein
ICD‐10‐CM	I82.B11	Acute embolism and thrombosis of right subclavian vein
ICD‐10‐CM	I82.B12	Acute embolism and thrombosis of left subclavian vein
ICD‐10‐CM	I82.B13	Acute embolism and thrombosis of subclavian vein, bilateral
ICD‐10‐CM	I82.B19	Acute embolism and thrombosis of unspecified subclavian vein
ICD‐10‐CM	I82.C11	Acute embolism and thrombosis of right internal jugular vein
ICD‐10‐CM	I82.C12	Acute embolism and thrombosis of left internal jugular vein
ICD‐10‐CM	I82.C13	Acute embolism and thrombosis of internal jugular vein, bilateral
ICD‐10‐CM	I82.C19	Acute embolism and thrombosis of unspecified internal jugular vein

Transient Ischemic Attack:

**Table:**

Code type	Code	Code description
ICD‐10‐CM	G45.0	Vertebro‐basilar artery syndrome
ICD‐10‐CM	G45.1	Carotid artery syndrome (hemispheric)
ICD‐10‐CM	G45.2	Multiple and bilateral precerebral artery syndromes
ICD‐10‐CM	G45.3	Amaurosis fugax
ICD‐10‐CM	G45.8	Other transient cerebral ischemic attacks and related syndromes
ICD‐10‐CM	G45.9	Transient cerebral ischemic attack, unspecified

Phrenic Nerve Damage:

**Table:**

Code type	Code	Code description
ICD‐10‐CM	J98.6	Disorders of diaphragm
ICD‐10‐CM	S14.9XXA	Injury of unspecified nerves of neck, initial encounter
ICD‐10‐CM	G97.81	Other intraoperative complications of nervous system
ICD‐10‐CM	G97.82	Other postprocedural complications and disorders of nervous system

Pericarditis:

**Table:**

Code type	Code	Code description
ICD‐10‐CM	I30.8	Other forms of acute pericarditis
ICD‐10‐CM	I30.9	Acute pericarditis, unspecified

Bleeding:

**Table:**

Code Type	Code	Code description
ICD‐10‐CM	I97.418	Intraoperative hemorrhage and hematoma of a circulatory system organ or structure complicating other circulatory system procedure
ICD‐10‐CM	I97.42	Intraoperative hemorrhage and hematoma of a circulatory system organ or structure complicating other procedure
ICD‐10‐CM	I97.618	Postprocedural hemorrhage and hematoma of a circulatory system organ or structure following other circulatory system procedure
ICD‐10‐CM	I97.620	Postprocedural hemorrhage of a circulatory system organ or structure following other procedure
ICD‐10‐CM	I97.621	Postprocedural hematoma of a circulatory system organ or structure following other procedure
ICD‐10‐CM	I97.638	Postprocedural hematoma of a circulatory system organ or structure following other circulatory system procedure
ICD‐10‐CM	I77.0	Arteriovenous fistula, acquired
ICD‐10‐CM	I97.89	Other postprocedural complications and disorders of the circulatory system, not elsewhere classified

Atrio‐Esophageal Fistula:

**Table:**

Code type	Code	Code description
ICD‐10‐CM	K22.3	Perforation of esophagus
ICD‐10‐CM	S27.812A	Contusion of esophagus (thoracic part), initial encounter
ICD‐10‐CM	S27.813A	Laceration of esophagus (thoracic part), initial encounter
ICD‐10‐CM	S27.818A	Other injury of esophagus (thoracic part), initial encounter
ICD‐10‐CM	S27.819A	Unspecified injury of esophagus (thoracic part), initial encounter

Severe Pulmonary Vein Stenosis Requiring Intervention:

**Table:**

Code type	Code	Code description
ICD‐10‐CM	S25.421A	Major laceration of right pulmonary blood vessels, initial encounter
ICD‐10‐CM	S25.422A	Major laceration of left pulmonary blood vessels, initial encounter
ICD‐10‐CM	S25.429A	Major laceration of unspecified pulmonary blood vessels, initial encounter

Vagal Nerve Injury:

**Table:**

Code type	Code	Code description
ICD‐10‐CM	S04.899A	Injury of other cranial nerves, unspecified side, initial encounter

Vascular Access Complication Requiring Repair:

**Table:**

Code type	Code	Code description
ICD‐10‐PCS	04CC3ZZ	Extirpation of Matter from Right Common Iliac Artery, Percutaneous Approach
ICD‐10‐PCS	04CD3ZZ	Extirpation of Matter from Left Common Iliac Artery, Percutaneous Approach
ICD‐10‐PCS	04CH3ZZ	Extirpation of Matter from Right External Iliac Artery, Percutaneous Approach
ICD‐10‐PCS	04CJ3ZZ	Extirpation of Matter from Left External Iliac Artery, Percutaneous Approach
ICD‐10‐PCS	04CK3ZZ	Extirpation of Matter from Right Femoral Artery, Percutaneous Approach
ICD‐10‐PCS	04CL3ZZ	Extirpation of Matter from Left Femoral Artery, Percutaneous Approach
ICD‐10‐PCS	04LC0DZ	Occlusion of Right Common Iliac Artery with Intraluminal Device, Open Approach
ICD‐10‐PCS	04LC3DZ	Occlusion of Right Common Iliac Artery with Intraluminal Device, Percutaneous Approach
ICD‐10‐PCS	04LC3ZZ	Occlusion of Right Common Iliac Artery, Percutaneous Approach
ICD‐10‐PCS	04LC4DZ	Occlusion of Right Common Iliac Artery with Intraluminal Device, Percutaneous Endoscopic Approach
ICD‐10‐PCS	04LD0DZ	Occlusion of Left Common Iliac Artery with Intraluminal Device, Open Approach
ICD‐10‐PCS	04LD3DZ	Occlusion of Left Common Iliac Artery with Intraluminal Device, Percutaneous Approach
ICD‐10‐PCS	04LD3ZZ	Occlusion of Left Common Iliac Artery, Percutaneous Approach
ICD‐10‐PCS	04LD4DZ	Occlusion of Left Common Iliac Artery with Intraluminal Device, Percutaneous Endoscopic Approach
ICD‐10‐PCS	04LH0DZ	Occlusion of Right External Iliac Artery with Intraluminal Device, Open Approach
ICD‐10‐PCS	04LH3DZ	Occlusion of Right External Iliac Artery with Intraluminal Device, Percutaneous Approach
ICD‐10‐PCS	04LH3ZZ	Occlusion of Right External Iliac Artery, Percutaneous Approach
ICD‐10‐PCS	04LH4DZ	Occlusion of Right External Iliac Artery with Intraluminal Device, Percutaneous Endoscopic Approach
ICD‐10‐PCS	04LJ0DZ	Occlusion of Left External Iliac Artery with Intraluminal Device, Open Approach
ICD‐10‐PCS	04LJ3DZ	Occlusion of Left External Iliac Artery with Intraluminal Device, Percutaneous Approach
ICD‐10‐PCS	04LJ3ZZ	Occlusion of Left External Iliac Artery, Percutaneous Approach
ICD‐10‐PCS	04LJ4DZ	Occlusion of Left External Iliac Artery with Intraluminal Device, Percutaneous Endoscopic Approach
ICD‐10‐PCS	04LK0DZ	Occlusion of Right Femoral Artery with Intraluminal Device, Open Approach
ICD‐10‐PCS	04LK3DZ	Occlusion of Right Femoral Artery with Intraluminal Device, Percutaneous Approach
ICD‐10‐PCS	04LK3ZZ	Occlusion of Right Femoral Artery, Percutaneous Approach
ICD‐10‐PCS	04LK4DZ	Occlusion of Right Femoral Artery with Intraluminal Device, Percutaneous Endoscopic Approach
ICD‐10‐PCS	04LL0DZ	Occlusion of Left Femoral Artery with Intraluminal Device, Open Approach
ICD‐10‐PCS	04LL3DZ	Occlusion of Left Femoral Artery with Intraluminal Device, Percutaneous Approach
ICD‐10‐PCS	04LL3ZZ	Occlusion of Left Femoral Artery, Percutaneous Approach
ICD‐10‐PCS	04LL4DZ	Occlusion of Left Femoral Artery with Intraluminal Device, Percutaneous Endoscopic Approach
ICD‐10‐PCS	04QC0ZZ	Repair Right Common Iliac Artery, Open Approach
ICD‐10‐PCS	04QC3ZZ	Repair Right Common Iliac Artery, Percutaneous Approach
ICD‐10‐PCS	04QC4ZZ	Repair Right Common Iliac Artery, Percutaneous Endoscopic Approach
ICD‐10‐PCS	04QD0ZZ	Repair Left Common Iliac Artery, Open Approach
ICD‐10‐PCS	04QD3ZZ	Repair Left Common Iliac Artery, Percutaneous Approach
ICD‐10‐PCS	04QD4ZZ	Repair Left Common Iliac Artery, Percutaneous Endoscopic Approach
ICD‐10‐PCS	04QH0ZZ	Repair Right External Iliac Artery, Open Approach
ICD‐10‐PCS	04QH3ZZ	Repair Right External Iliac Artery, Percutaneous Approach
ICD‐10‐PCS	04QH4ZZ	Repair Right External Iliac Artery, Percutaneous Endoscopic Approach
ICD‐10‐PCS	04QJ0ZZ	Repair Left External Iliac Artery, Open Approach
ICD‐10‐PCS	04QJ3ZZ	Repair Left External Iliac Artery, Percutaneous Approach
ICD‐10‐PCS	04QJ4ZZ	Repair Left External Iliac Artery, Percutaneous Endoscopic Approach
ICD‐10‐PCS	04QK0ZZ	Repair Right Femoral Artery, Open Approach
ICD‐10‐PCS	04QK3ZZ	Repair Right Femoral Artery, Percutaneous Approach
ICD‐10‐PCS	04QK4ZZ	Repair Right Femoral Artery, Percutaneous Endoscopic Approach
ICD‐10‐PCS	04QL0ZZ	Repair Left Femoral Artery, Open Approach
ICD‐10‐PCS	04QL3ZZ	Repair Left Femoral Artery, Percutaneous Approach
ICD‐10‐PCS	04QL4ZZ	Repair Left Femoral Artery, Percutaneous Endoscopic Approach
ICD‐10‐PCS	04SC0ZZ	Reposition Right Common Iliac Artery, Open Approach
ICD‐10‐PCS	04SC3ZZ	Reposition Right Common Iliac Artery, Percutaneous Approach
ICD‐10‐PCS	04SC4ZZ	Reposition Right Common Iliac Artery, Percutaneous Endoscopic Approach
ICD‐10‐PCS	04SD0ZZ	Reposition Left Common Iliac Artery, Open Approach
ICD‐10‐PCS	04SD3ZZ	Reposition Left Common Iliac Artery, Percutaneous Approach
ICD‐10‐PCS	04SD4ZZ	Reposition Left Common Iliac Artery, Percutaneous Endoscopic Approach
ICD‐10‐PCS	04SH0ZZ	Reposition Right External Iliac Artery, Open Approach
ICD‐10‐PCS	04SH3ZZ	Reposition Right External Iliac Artery, Percutaneous Approach
ICD‐10‐PCS	04SH4ZZ	Reposition Right External Iliac Artery, Percutaneous Endoscopic Approach
ICD‐10‐PCS	04SJ0ZZ	Reposition Left External Iliac Artery, Open Approach
ICD‐10‐PCS	04SJ3ZZ	Reposition Left External Iliac Artery, Percutaneous Approach
ICD‐10‐PCS	04SJ4ZZ	Reposition Left External Iliac Artery, Percutaneous Endoscopic Approach
ICD‐10‐PCS	04SK0ZZ	Reposition Right Femoral Artery, Open Approach
ICD‐10‐PCS	04SK3ZZ	Reposition Right Femoral Artery, Percutaneous Approach
ICD‐10‐PCS	04SK4ZZ	Reposition Right Femoral Artery, Percutaneous Endoscopic Approach
ICD‐10‐PCS	04SL0ZZ	Reposition Left Femoral Artery, Open Approach
ICD‐10‐PCS	04SL3ZZ	Reposition Left Femoral Artery, Percutaneous Approach
ICD‐10‐PCS	04SL4ZZ	Reposition Left Femoral Artery, Percutaneous Endoscopic Approach
ICD‐10‐PCS	04UC07Z	Supplement Right Common Iliac Artery with Autologous Tissue Substitute, Open Approach
ICD‐10‐PCS	04UC0JZ	Supplement Right Common Iliac Artery with Synthetic Substitute, Open Approach
ICD‐10‐PCS	04UC0KZ	Supplement Right Common Iliac Artery with Nonautologous Tissue Substitute, Open Approach
ICD‐10‐PCS	04UC37Z	Supplement Right Common Iliac Artery with Autologous Tissue Substitute, Percutaneous Approach
ICD‐10‐PCS	04UC3JZ	Supplement Right Common Iliac Artery with Synthetic Substitute, Percutaneous Approach
ICD‐10‐PCS	04UC3KZ	Supplement Right Common Iliac Artery with Nonautologous Tissue Substitute, Percutaneous Approach
ICD‐10‐PCS	04UC47Z	Supplement Right Common Iliac Artery with Autologous Tissue Substitute, Percutaneous Endoscopic Approach
ICD‐10‐PCS	04UC4JZ	Supplement Right Common Iliac Artery with Synthetic Substitute, Percutaneous Endoscopic Approach
ICD‐10‐PCS	04UC4KZ	Supplement Right Common Iliac Artery with Nonautologous Tissue Substitute, Percutaneous Endoscopic Approach
ICD‐10‐PCS	04UD07Z	Supplement Left Common Iliac Artery with Autologous Tissue Substitute, Open Approach
ICD‐10‐PCS	04UD0JZ	Supplement Left Common Iliac Artery with Synthetic Substitute, Open Approach
ICD‐10‐PCS	04UD0KZ	Supplement Left Common Iliac Artery with Nonautologous Tissue Substitute, Open Approach
ICD‐10‐PCS	04UD37Z	Supplement Left Common Iliac Artery with Autologous Tissue Substitute, Percutaneous Approach
ICD‐10‐PCS	04UD3JZ	Supplement Left Common Iliac Artery with Synthetic Substitute, Percutaneous Approach
ICD‐10‐PCS	04UD3KZ	Supplement Left Common Iliac Artery with Nonautologous Tissue Substitute, Percutaneous Approach
ICD‐10‐PCS	04UD47Z	Supplement Left Common Iliac Artery with Autologous Tissue Substitute, Percutaneous Endoscopic Approach
ICD‐10‐PCS	04UD4JZ	Supplement Left Common Iliac Artery with Synthetic Substitute, Percutaneous Endoscopic Approach
ICD‐10‐PCS	04UD4KZ	Supplement Left Common Iliac Artery with Nonautologous Tissue Substitute, Percutaneous Endoscopic Approach
ICD‐10‐PCS	04UH07Z	Supplement Right External Iliac Artery with Autologous Tissue Substitute, Open Approach
ICD‐10‐PCS	04UH0JZ	Supplement Right External Iliac Artery with Synthetic Substitute, Open Approach
ICD‐10‐PCS	04UH0KZ	Supplement Right External Iliac Artery with Nonautologous Tissue Substitute, Open Approach
ICD‐10‐PCS	04UH37Z	Supplement Right External Iliac Artery with Autologous Tissue Substitute, Percutaneous Approach
ICD‐10‐PCS	04UH3JZ	Supplement Right External Iliac Artery with Synthetic Substitute, Percutaneous Approach
ICD‐10‐PCS	04UH3KZ	Supplement Right External Iliac Artery with Nonautologous Tissue Substitute, Percutaneous Approach
ICD‐10‐PCS	04UH47Z	Supplement Right External Iliac Artery with Autologous Tissue Substitute, Percutaneous Endoscopic Approach
ICD‐10‐PCS	04UH4JZ	Supplement Right External Iliac Artery with Synthetic Substitute, Percutaneous Endoscopic Approach
ICD‐10‐PCS	04UH4KZ	Supplement Right External Iliac Artery with Nonautologous Tissue Substitute, Percutaneous Endoscopic Approach
ICD‐10‐PCS	04UJ07Z	Supplement Left External Iliac Artery with Autologous Tissue Substitute, Open Approach
ICD‐10‐PCS	04UJ0JZ	Supplement Left External Iliac Artery with Synthetic Substitute, Open Approach
ICD‐10‐PCS	04UJ0KZ	Supplement Left External Iliac Artery with Nonautologous Tissue Substitute, Open Approach
ICD‐10‐PCS	04UJ37Z	Supplement Left External Iliac Artery with Autologous Tissue Substitute, Percutaneous Approach
ICD‐10‐PCS	04UJ3JZ	Supplement Left External Iliac Artery with Synthetic Substitute, Percutaneous Approach
ICD‐10‐PCS	04UJ3KZ	Supplement Left External Iliac Artery with Nonautologous Tissue Substitute, Percutaneous Approach
ICD‐10‐PCS	04UJ47Z	Supplement Left External Iliac Artery with Autologous Tissue Substitute, Percutaneous Endoscopic Approach
ICD‐10‐PCS	04UJ4JZ	Supplement Left External Iliac Artery with Synthetic Substitute, Percutaneous Endoscopic Approach
ICD‐10‐PCS	04UJ4KZ	Supplement Left External Iliac Artery with Nonautologous Tissue Substitute, Percutaneous Endoscopic Approach
ICD‐10‐PCS	04UK07Z	Supplement Right Femoral Artery with Autologous Tissue Substitute, Open Approach
ICD‐10‐PCS	04UK0JZ	Supplement Right Femoral Artery with Synthetic Substitute, Open Approach
ICD‐10‐PCS	04UK0KZ	Supplement Right Femoral Artery with Nonautologous Tissue Substitute, Open Approach
ICD‐10‐PCS	04UK37Z	Supplement Right Femoral Artery with Autologous Tissue Substitute, Percutaneous Approach
ICD‐10‐PCS	04UK3JZ	Supplement Right Femoral Artery with Synthetic Substitute, Percutaneous Approach
ICD‐10‐PCS	04UK3KZ	Supplement Right Femoral Artery with Nonautologous Tissue Substitute, Percutaneous Approach
ICD‐10‐PCS	04UK47Z	Supplement Right Femoral Artery with Autologous Tissue Substitute, Percutaneous Endoscopic Approach
ICD‐10‐PCS	04UK4JZ	Supplement Right Femoral Artery with Synthetic Substitute, Percutaneous Endoscopic Approach
ICD‐10‐PCS	04UK4KZ	Supplement Right Femoral Artery with Nonautologous Tissue Substitute, Percutaneous Endoscopic Approach
ICD‐10‐PCS	04UL07Z	Supplement Left Femoral Artery with Autologous Tissue Substitute, Open Approach
ICD‐10‐PCS	04UL0JZ	Supplement Left Femoral Artery with Synthetic Substitute, Open Approach
ICD‐10‐PCS	04UL0KZ	Supplement Left Femoral Artery with Nonautologous Tissue Substitute, Open Approach
ICD‐10‐PCS	04UL37Z	Supplement Left Femoral Artery with Autologous Tissue Substitute, Percutaneous Approach
ICD‐10‐PCS	04UL3JZ	Supplement Left Femoral Artery with Synthetic Substitute, Percutaneous Approach
ICD‐10‐PCS	04UL3KZ	Supplement Left Femoral Artery with Nonautologous Tissue Substitute, Percutaneous Approach
ICD‐10‐PCS	04UL47Z	Supplement Left Femoral Artery with Autologous Tissue Substitute, Percutaneous Endoscopic Approach
ICD‐10‐PCS	04UL4JZ	Supplement Left Femoral Artery with Synthetic Substitute, Percutaneous Endoscopic Approach
ICD‐10‐PCS	04UL4KZ	Supplement Left Femoral Artery with Nonautologous Tissue Substitute, Percutaneous Endoscopic Approach
ICD‐10‐PCS	04VC0DZ	Restriction of Right Common Iliac Artery with Intraluminal Device, Open Approach
ICD‐10‐PCS	04VC0EZ	Restriction of Right Common Iliac Artery with Branched or Fenestrated Intraluminal Device, One or Two Arteries, Open Approach
ICD‐10‐PCS	04VC0ZZ	Restriction of Right Common Iliac Artery, Open Approach
ICD‐10‐PCS	04VC3DZ	Restriction of Right Common Iliac Artery with Intraluminal Device, Percutaneous Approach
ICD‐10‐PCS	04VC3EZ	Restriction of Right Common Iliac Artery with Branched or Fenestrated Intraluminal Device, One or Two Arteries, Percutaneous Approach
ICD‐10‐PCS	04VC3ZZ	Restriction of Right Common Iliac Artery, Percutaneous Approach
ICD‐10‐PCS	04VC4DZ	Restriction of Right Common Iliac Artery with Intraluminal Device, Percutaneous Endoscopic Approach
ICD‐10‐PCS	04VC4EZ	Restriction of Right Common Iliac Artery with Branched or Fenestrated Intraluminal Device, One or Two Arteries, Percutaneous Endoscopic Approach
ICD‐10‐PCS	04VC4ZZ	Restriction of Right Common Iliac Artery, Percutaneous Endoscopic Approach
ICD‐10‐PCS	04VD0DZ	Restriction of Left Common Iliac Artery with Intraluminal Device, Open Approach
ICD‐10‐PCS	04VD0EZ	Restriction of Left Common Iliac Artery with Branched or Fenestrated Intraluminal Device, One or Two Arteries, Open Approach
ICD‐10‐PCS	04VD0ZZ	Restriction of Left Common Iliac Artery, Open Approach
ICD‐10‐PCS	04VD3DZ	Restriction of Left Common Iliac Artery with Intraluminal Device, Percutaneous Approach
ICD‐10‐PCS	04VD3EZ	Restriction of Left Common Iliac Artery with Branched or Fenestrated Intraluminal Device, One or Two Arteries, Percutaneous Approach
ICD‐10‐PCS	04VD3ZZ	Restriction of Left Common Iliac Artery, Percutaneous Approach
ICD‐10‐PCS	04VD4DZ	Restriction of Left Common Iliac Artery with Intraluminal Device, Percutaneous Endoscopic Approach
ICD‐10‐PCS	04VD4EZ	Restriction of Left Common Iliac Artery with Branched or Fenestrated Intraluminal Device, One or Two Arteries, Percutaneous Endoscopic Approach
ICD‐10‐PCS	04VD4ZZ	Restriction of Left Common Iliac Artery, Percutaneous Endoscopic Approach
ICD‐10‐PCS	04VH0DZ	Restriction of Right External Iliac Artery with Intraluminal Device, Open Approach
ICD‐10‐PCS	04VH0ZZ	Restriction of Right External Iliac Artery, Open Approach
ICD‐10‐PCS	04VH3DZ	Restriction of Right External Iliac Artery with Intraluminal Device, Percutaneous Approach
ICD‐10‐PCS	04VH3ZZ	Restriction of Right External Iliac Artery, Percutaneous Approach
ICD‐10‐PCS	04VH4DZ	Restriction of Right External Iliac Artery with Intraluminal Device, Percutaneous Endoscopic Approach
ICD‐10‐PCS	04VH4ZZ	Restriction of Right External Iliac Artery, Percutaneous Endoscopic Approach
ICD‐10‐PCS	04VJ0DZ	Restriction of Left External Iliac Artery with Intraluminal Device, Open Approach
ICD‐10‐PCS	04VJ0ZZ	Restriction of Left External Iliac Artery, Open Approach
ICD‐10‐PCS	04VJ3DZ	Restriction of Left External Iliac Artery with Intraluminal Device, Percutaneous Approach
ICD‐10‐PCS	04VJ3ZZ	Restriction of Left External Iliac Artery, Percutaneous Approach
ICD‐10‐PCS	04VJ4DZ	Restriction of Left External Iliac Artery with Intraluminal Device, Percutaneous Endoscopic Approach
ICD‐10‐PCS	04VJ4ZZ	Restriction of Left External Iliac Artery, Percutaneous Endoscopic Approach
ICD‐10‐PCS	04VK0DZ	Restriction of Right Femoral Artery with Intraluminal Device, Open Approach
ICD‐10‐PCS	04VK0ZZ	Restriction of Right Femoral Artery, Open Approach
ICD‐10‐PCS	04VK3DZ	Restriction of Right Femoral Artery with Intraluminal Device, Percutaneous Approach
ICD‐10‐PCS	04VK3ZZ	Restriction of Right Femoral Artery, Percutaneous Approach
ICD‐10‐PCS	04VK4DZ	Restriction of Right Femoral Artery with Intraluminal Device, Percutaneous Endoscopic Approach
ICD‐10‐PCS	04VK4ZZ	Restriction of Right Femoral Artery, Percutaneous Endoscopic Approach
ICD‐10‐PCS	04VL0DZ	Restriction of Left Femoral Artery with Intraluminal Device, Open Approach
ICD‐10‐PCS	04VL0ZZ	Restriction of Left Femoral Artery, Open Approach
ICD‐10‐PCS	04VL3DZ	Restriction of Left Femoral Artery with Intraluminal Device, Percutaneous Approach
ICD‐10‐PCS	04VL3ZZ	Restriction of Left Femoral Artery, Percutaneous Approach
ICD‐10‐PCS	04VL4DZ	Restriction of Left Femoral Artery with Intraluminal Device, Percutaneous Endoscopic Approach
ICD‐10‐PCS	04VL4ZZ	Restriction of Left Femoral Artery, Percutaneous Endoscopic Approach
ICD‐10‐PCS	06CC3ZZ	Extirpation of Matter from Right Common Iliac Vein, Percutaneous Approach
ICD‐10‐PCS	06CD3ZZ	Extirpation of Matter from Left Common Iliac Vein, Percutaneous Approach
ICD‐10‐PCS	06CF3ZZ	Extirpation of Matter from Right External Iliac Vein, Percutaneous Approach
ICD‐10‐PCS	06CG3ZZ	Extirpation of Matter from Left External Iliac Vein, Percutaneous Approach
ICD‐10‐PCS	06CM3ZZ	Extirpation of Matter from Right Femoral Vein, Percutaneous Approach
ICD‐10‐PCS	06CN3ZZ	Extirpation of Matter from Left Femoral Vein, Percutaneous Approach
ICD‐10‐PCS	06LC0DZ	Occlusion of Right Common Iliac Vein with Intraluminal Device, Open Approach
ICD‐10‐PCS	06LC3DZ	Occlusion of Right Common Iliac Vein with Intraluminal Device, Percutaneous Approach
ICD‐10‐PCS	06LC3ZZ	Occlusion of Right Common Iliac Vein, Percutaneous Approach
ICD‐10‐PCS	06LC4DZ	Occlusion of Right Common Iliac Vein with Intraluminal Device, Percutaneous Endoscopic Approach
ICD‐10‐PCS	06LD0DZ	Occlusion of Left Common Iliac Vein with Intraluminal Device, Open Approach
ICD‐10‐PCS	06LD3DZ	Occlusion of Left Common Iliac Vein with Intraluminal Device, Percutaneous Approach
ICD‐10‐PCS	06LD3ZZ	Occlusion of Left Common Iliac Vein, Percutaneous Approach
ICD‐10‐PCS	06LD4DZ	Occlusion of Left Common Iliac Vein with Intraluminal Device, Percutaneous Endoscopic Approach
ICD‐10‐PCS	06LF0DZ	Occlusion of Right External Iliac Vein with Intraluminal Device, Open Approach
ICD‐10‐PCS	06LF3DZ	Occlusion of Right External Iliac Vein with Intraluminal Device, Percutaneous Approach
ICD‐10‐PCS	06LF3ZZ	Occlusion of Right External Iliac Vein, Percutaneous Approach
ICD‐10‐PCS	06LF4DZ	Occlusion of Right External Iliac Vein with Intraluminal Device, Percutaneous Endoscopic Approach
ICD‐10‐PCS	06LG0DZ	Occlusion of Left External Iliac Vein with Intraluminal Device, Open Approach
ICD‐10‐PCS	06LG3DZ	Occlusion of Left External Iliac Vein with Intraluminal Device, Percutaneous Approach
ICD‐10‐PCS	06LG3ZZ	Occlusion of Left External Iliac Vein, Percutaneous Approach
ICD‐10‐PCS	06LG4DZ	Occlusion of Left External Iliac Vein with Intraluminal Device, Percutaneous Endoscopic Approach
ICD‐10‐PCS	06LM0DZ	Occlusion of Right Femoral Vein with Intraluminal Device, Open Approach
ICD‐10‐PCS	06LM3DZ	Occlusion of Right Femoral Vein with Intraluminal Device, Percutaneous Approach
ICD‐10‐PCS	06LM3ZZ	Occlusion of Right Femoral Vein, Percutaneous Approach
ICD‐10‐PCS	06LM4DZ	Occlusion of Right Femoral Vein with Intraluminal Device, Percutaneous Endoscopic Approach
ICD‐10‐PCS	06LN0DZ	Occlusion of Left Femoral Vein with Intraluminal Device, Open Approach
ICD‐10‐PCS	06LN3DZ	Occlusion of Left Femoral Vein with Intraluminal Device, Percutaneous Approach
ICD‐10‐PCS	06LN3ZZ	Occlusion of Left Femoral Vein, Percutaneous Approach
ICD‐10‐PCS	06LN4DZ	Occlusion of Left Femoral Vein with Intraluminal Device, Percutaneous Endoscopic Approach
ICD‐10‐PCS	06SC0ZZ	Reposition Right Common Iliac Vein, Open Approach
ICD‐10‐PCS	06SC3ZZ	Reposition Right Common Iliac Vein, Percutaneous Approach
ICD‐10‐PCS	06SC4ZZ	Reposition Right Common Iliac Vein, Percutaneous Endoscopic Approach
ICD‐10‐PCS	06SD0ZZ	Reposition Left Common Iliac Vein, Open Approach
ICD‐10‐PCS	06SD3ZZ	Reposition Left Common Iliac Vein, Percutaneous Approach
ICD‐10‐PCS	06SD4ZZ	Reposition Left Common Iliac Vein, Percutaneous Endoscopic Approach
ICD‐10‐PCS	06SF0ZZ	Reposition Right External Iliac Vein, Open Approach
ICD‐10‐PCS	06SF3ZZ	Reposition Right External Iliac Vein, Percutaneous Approach
ICD‐10‐PCS	06SF4ZZ	Reposition Right External Iliac Vein, Percutaneous Endoscopic Approach
ICD‐10‐PCS	06SG0ZZ	Reposition Left External Iliac Vein, Open Approach
ICD‐10‐PCS	06SG3ZZ	Reposition Left External Iliac Vein, Percutaneous Approach
ICD‐10‐PCS	06SG4ZZ	Reposition Left External Iliac Vein, Percutaneous Endoscopic Approach
ICD‐10‐PCS	06SM0ZZ	Reposition Right Femoral Vein, Open Approach
ICD‐10‐PCS	06SM3ZZ	Reposition Right Femoral Vein, Percutaneous Approach
ICD‐10‐PCS	06SM4ZZ	Reposition Right Femoral Vein, Percutaneous Endoscopic Approach
ICD‐10‐PCS	06SN0ZZ	Reposition Left Femoral Vein, Open Approach
ICD‐10‐PCS	06SN3ZZ	Reposition Left Femoral Vein, Percutaneous Approach
ICD‐10‐PCS	06SN4ZZ	Reposition Left Femoral Vein, Percutaneous Endoscopic Approach
ICD‐10‐PCS	06UC07Z	Supplement Right Common Iliac Vein with Autologous Tissue Substitute, Open Approach
ICD‐10‐PCS	06UC0JZ	Supplement Right Common Iliac Vein with Synthetic Substitute, Open Approach
ICD‐10‐PCS	06UC0KZ	Supplement Right Common Iliac Vein with Nonautologous Tissue Substitute, Open Approach
ICD‐10‐PCS	06UC37Z	Supplement Right Common Iliac Vein with Autologous Tissue Substitute, Percutaneous Approach
ICD‐10‐PCS	06UC3JZ	Supplement Right Common Iliac Vein with Synthetic Substitute, Percutaneous Approach
ICD‐10‐PCS	06UC3KZ	Supplement Right Common Iliac Vein with Nonautologous Tissue Substitute, Percutaneous Approach
ICD‐10‐PCS	06UC47Z	Supplement Right Common Iliac Vein with Autologous Tissue Substitute, Percutaneous Endoscopic Approach
ICD‐10‐PCS	06UC4JZ	Supplement Right Common Iliac Vein with Synthetic Substitute, Percutaneous Endoscopic Approach
ICD‐10‐PCS	06UC4KZ	Supplement Right Common Iliac Vein with Nonautologous Tissue Substitute, Percutaneous Endoscopic Approach
ICD‐10‐PCS	06UD07Z	Supplement Left Common Iliac Vein with Autologous Tissue Substitute, Open Approach
ICD‐10‐PCS	06UD0JZ	Supplement Left Common Iliac Vein with Synthetic Substitute, Open Approach
ICD‐10‐PCS	06UD0KZ	Supplement Left Common Iliac Vein with Nonautologous Tissue Substitute, Open Approach
ICD‐10‐PCS	06UD37Z	Supplement Left Common Iliac Vein with Autologous Tissue Substitute, Percutaneous Approach
ICD‐10‐PCS	06UD3JZ	Supplement Left Common Iliac Vein with Synthetic Substitute, Percutaneous Approach
ICD‐10‐PCS	06UD3KZ	Supplement Left Common Iliac Vein with Nonautologous Tissue Substitute, Percutaneous Approach
ICD‐10‐PCS	06UD47Z	Supplement Left Common Iliac Vein with Autologous Tissue Substitute, Percutaneous Endoscopic Approach
ICD‐10‐PCS	06UD4JZ	Supplement Left Common Iliac Vein with Synthetic Substitute, Percutaneous Endoscopic Approach
ICD‐10‐PCS	06UD4KZ	Supplement Left Common Iliac Vein with Nonautologous Tissue Substitute, Percutaneous Endoscopic Approach
ICD‐10‐PCS	06UF07Z	Supplement Right External Iliac Vein with Autologous Tissue Substitute, Open Approach
ICD‐10‐PCS	06UF0JZ	Supplement Right External Iliac Vein with Synthetic Substitute, Open Approach
ICD‐10‐PCS	06UF0KZ	Supplement Right External Iliac Vein with Nonautologous Tissue Substitute, Open Approach
ICD‐10‐PCS	06UF37Z	Supplement Right External Iliac Vein with Autologous Tissue Substitute, Percutaneous Approach
ICD‐10‐PCS	06UF3JZ	Supplement Right External Iliac Vein with Synthetic Substitute, Percutaneous Approach
ICD‐10‐PCS	06UF3KZ	Supplement Right External Iliac Vein with Nonautologous Tissue Substitute, Percutaneous Approach
ICD‐10‐PCS	06UF47Z	Supplement Right External Iliac Vein with Autologous Tissue Substitute, Percutaneous Endoscopic Approach
ICD‐10‐PCS	06UF4JZ	Supplement Right External Iliac Vein with Synthetic Substitute, Percutaneous Endoscopic Approach
ICD‐10‐PCS	06UF4KZ	Supplement Right External Iliac Vein with Nonautologous Tissue Substitute, Percutaneous Endoscopic Approach
ICD‐10‐PCS	06UG07Z	Supplement Left External Iliac Vein with Autologous Tissue Substitute, Open Approach
ICD‐10‐PCS	06UG0JZ	Supplement Left External Iliac Vein with Synthetic Substitute, Open Approach
ICD‐10‐PCS	06UG0KZ	Supplement Left External Iliac Vein with Nonautologous Tissue Substitute, Open Approach
ICD‐10‐PCS	06UG37Z	Supplement Left External Iliac Vein with Autologous Tissue Substitute, Percutaneous Approach
ICD‐10‐PCS	06UG3JZ	Supplement Left External Iliac Vein with Synthetic Substitute, Percutaneous Approach
ICD‐10‐PCS	06UG3KZ	Supplement Left External Iliac Vein with Nonautologous Tissue Substitute, Percutaneous Approach
ICD‐10‐PCS	06UG47Z	Supplement Left External Iliac Vein with Autologous Tissue Substitute, Percutaneous Endoscopic Approach
ICD‐10‐PCS	06UG4JZ	Supplement Left External Iliac Vein with Synthetic Substitute, Percutaneous Endoscopic Approach
ICD‐10‐PCS	06UG4KZ	Supplement Left External Iliac Vein with Nonautologous Tissue Substitute, Percutaneous Endoscopic Approach
ICD‐10‐PCS	06UM07Z	Supplement Right Femoral Vein with Autologous Tissue Substitute, Open Approach
ICD‐10‐PCS	06UM0JZ	Supplement Right Femoral Vein with Synthetic Substitute, Open Approach
ICD‐10‐PCS	06UM0KZ	Supplement Right Femoral Vein with Nonautologous Tissue Substitute, Open Approach
ICD‐10‐PCS	06UM37Z	Supplement Right Femoral Vein with Autologous Tissue Substitute, Percutaneous Approach
ICD‐10‐PCS	06UM3JZ	Supplement Right Femoral Vein with Synthetic Substitute, Percutaneous Approach
ICD‐10‐PCS	06UM3KZ	Supplement Right Femoral Vein with Nonautologous Tissue Substitute, Percutaneous Approach
ICD‐10‐PCS	06UM47Z	Supplement Right Femoral Vein with Autologous Tissue Substitute, Percutaneous Endoscopic Approach
ICD‐10‐PCS	06UM4JZ	Supplement Right Femoral Vein with Synthetic Substitute, Percutaneous Endoscopic Approach
ICD‐10‐PCS	06UM4KZ	Supplement Right Femoral Vein with Nonautologous Tissue Substitute, Percutaneous Endoscopic Approach
ICD‐10‐PCS	06UN07Z	Supplement Left Femoral Vein with Autologous Tissue Substitute, Open Approach
ICD‐10‐PCS	06UN0JZ	Supplement Left Femoral Vein with Synthetic Substitute, Open Approach
ICD‐10‐PCS	06UN0KZ	Supplement Left Femoral Vein with Nonautologous Tissue Substitute, Open Approach
ICD‐10‐PCS	06UN37Z	Supplement Left Femoral Vein with Autologous Tissue Substitute, Percutaneous Approach
ICD‐10‐PCS	06UN3JZ	Supplement Left Femoral Vein with Synthetic Substitute, Percutaneous Approach
ICD‐10‐PCS	06UN3KZ	Supplement Left Femoral Vein with Nonautologous Tissue Substitute, Percutaneous Approach
ICD‐10‐PCS	06UN47Z	Supplement Left Femoral Vein with Autologous Tissue Substitute, Percutaneous Endoscopic Approach
ICD‐10‐PCS	06UN4JZ	Supplement Left Femoral Vein with Synthetic Substitute, Percutaneous Endoscopic Approach
ICD‐10‐PCS	06UN4KZ	Supplement Left Femoral Vein with Nonautologous Tissue Substitute, Percutaneous Endoscopic Approach
ICD‐10‐PCS	06VC0DZ	Restriction of Right Common Iliac Vein with Intraluminal Device, Open Approach
ICD‐10‐PCS	06VC0ZZ	Restriction of Right Common Iliac Vein, Open Approach
ICD‐10‐PCS	06VC3DZ	Restriction of Right Common Iliac Vein with Intraluminal Device, Percutaneous Approach
ICD‐10‐PCS	06VC3ZZ	Restriction of Right Common Iliac Vein, Percutaneous Approach
ICD‐10‐PCS	06VC4DZ	Restriction of Right Common Iliac Vein with Intraluminal Device, Percutaneous Endoscopic Approach
ICD‐10‐PCS	06VC4ZZ	Restriction of Right Common Iliac Vein, Percutaneous Endoscopic Approach
ICD‐10‐PCS	06VD0DZ	Restriction of Left Common Iliac Vein with Intraluminal Device, Open Approach
ICD‐10‐PCS	06VD0ZZ	Restriction of Left Common Iliac Vein, Open Approach
ICD‐10‐PCS	06VD3DZ	Restriction of Left Common Iliac Vein with Intraluminal Device, Percutaneous Approach
ICD‐10‐PCS	06VD3ZZ	Restriction of Left Common Iliac Vein, Percutaneous Approach
ICD‐10‐PCS	06VD4DZ	Restriction of Left Common Iliac Vein with Intraluminal Device, Percutaneous Endoscopic Approach
ICD‐10‐PCS	06VD4ZZ	Restriction of Left Common Iliac Vein, Percutaneous Endoscopic Approach
ICD‐10‐PCS	06VF0DZ	Restriction of Right External Iliac Vein with Intraluminal Device, Open Approach
ICD‐10‐PCS	06VF0ZZ	Restriction of Right External Iliac Vein, Open Approach
ICD‐10‐PCS	06VF3DZ	Restriction of Right External Iliac Vein with Intraluminal Device, Percutaneous Approach
ICD‐10‐PCS	06VF3ZZ	Restriction of Right External Iliac Vein, Percutaneous Approach
ICD‐10‐PCS	06VF4DZ	Restriction of Right External Iliac Vein with Intraluminal Device, Percutaneous Endoscopic Approach
ICD‐10‐PCS	06VF4ZZ	Restriction of Right External Iliac Vein, Percutaneous Endoscopic Approach
ICD‐10‐PCS	06VG0DZ	Restriction of Left External Iliac Vein with Intraluminal Device, Open Approach
ICD‐10‐PCS	06VG0ZZ	Restriction of Left External Iliac Vein, Open Approach
ICD‐10‐PCS	06VG3DZ	Restriction of Left External Iliac Vein with Intraluminal Device, Percutaneous Approach
ICD‐10‐PCS	06VG3ZZ	Restriction of Left External Iliac Vein, Percutaneous Approach
ICD‐10‐PCS	06VG4DZ	Restriction of Left External Iliac Vein with Intraluminal Device, Percutaneous Endoscopic Approach
ICD‐10‐PCS	06VG4ZZ	Restriction of Left External Iliac Vein, Percutaneous Endoscopic Approach
ICD‐10‐PCS	06VM0DZ	Restriction of Right Femoral Vein with Intraluminal Device, Open Approach
ICD‐10‐PCS	06VM0ZZ	Restriction of Right Femoral Vein, Open Approach
ICD‐10‐PCS	06VM3DZ	Restriction of Right Femoral Vein with Intraluminal Device, Percutaneous Approach
ICD‐10‐PCS	06VM3ZZ	Restriction of Right Femoral Vein, Percutaneous Approach
ICD‐10‐PCS	06VM4DZ	Restriction of Right Femoral Vein with Intraluminal Device, Percutaneous Endoscopic Approach
ICD‐10‐PCS	06VM4ZZ	Restriction of Right Femoral Vein, Percutaneous Endoscopic Approach
ICD‐10‐PCS	06VN0DZ	Restriction of Left Femoral Vein with Intraluminal Device, Open Approach
ICD‐10‐PCS	06VN0ZZ	Restriction of Left Femoral Vein, Open Approach
ICD‐10‐PCS	06VN3DZ	Restriction of Left Femoral Vein with Intraluminal Device, Percutaneous Approach
ICD‐10‐PCS	06VN3ZZ	Restriction of Left Femoral Vein, Percutaneous Approach
ICD‐10‐PCS	06VN4DZ	Restriction of Left Femoral Vein with Intraluminal Device, Percutaneous Endoscopic Approach
ICD‐10‐PCS	06VN4ZZ	Restriction of Left Femoral Vein, Percutaneous Endoscopic Approach
CPT	35211	Repair blood vessel, direct; intrathoracic, with bypass
CPT	35216	Repair blood vessel, direct; intrathoracic, without bypass
CPT	35226	Repair blood vessel, direct; lower extremity
CPT	35246	Repair blood vessel with vein graft; intrathoracic, without bypass
CPT	35251	Repair blood vessel with vein graft; intra‐abdominal
CPT	35256	Repair blood vessel with vein graft; lower extremity
CPT	35276	Repair blood vessel with graft other than vein; intrathoracic, without bypass
CPT	35281	Repair blood vessel with graft other than vein; intra‐abdominal
CPT	35286	Repair blood vessel with graft other than vein; lower extremity

Blood Transfusion:

**Table:**

Code type	Code	Code description
ICD‐10‐PCS	30230H1	Transfusion of Nonautologous Whole Blood into Peripheral Vein, Open Approach
ICD‐10‐PCS	30230N1	Transfusion of Nonautologous Red Blood Cells into Peripheral Vein, Open Approach
ICD‐10‐PCS	30230P1	Transfusion of Nonautologous Frozen Red Cells into Peripheral Vein, Open Approach
ICD‐10‐PCS	30233H1	Transfusion of Nonautologous Whole Blood into Peripheral Vein, Percutaneous Approach
ICD‐10‐PCS	30233N1	Transfusion of Nonautologous Red Blood Cells into Peripheral Vein, Percutaneous Approach
ICD‐10‐PCS	30233P1	Transfusion of Nonautologous Frozen Red Cells into Peripheral Vein, Percutaneous Approach
ICD‐10‐PCS	30240H1	Transfusion of Nonautologous Whole Blood into Central Vein, Open Approach
ICD‐10‐PCS	30240N1	Transfusion of Nonautologous Red Blood Cells into Central Vein, Open Approach
ICD‐10‐PCS	30240P1	Transfusion of Nonautologous Frozen Red Cells into Central Vein, Open Approach
ICD‐10‐PCS	30243H1	Transfusion of Nonautologous Whole Blood into Central Vein, Percutaneous Approach
ICD‐10‐PCS	30243N1	Transfusion of Nonautologous Red Blood Cells into Central Vein, Percutaneous Approach
ICD‐10‐PCS	30243P1	Transfusion of Nonautologous Frozen Red Cells into Central Vein, Percutaneous Approach
ICD‐10‐PCS	30250H1	Transfusion of Nonautologous Whole Blood into Peripheral Artery, Open Approach
ICD‐10‐PCS	30250N1	Transfusion of Nonautologous Red Blood Cells into Peripheral Artery, Open Approach
ICD‐10‐PCS	30250P1	Transfusion of Nonautologous Frozen Red Cells into Peripheral Artery, Open Approach
ICD‐10‐PCS	30253H1	Transfusion of Nonautologous Whole Blood into Peripheral Artery, Percutaneous Approach
ICD‐10‐PCS	30253N1	Transfusion of Nonautologous Red Blood Cells into Peripheral Artery, Percutaneous Approach
ICD‐10‐PCS	30253P1	Transfusion of Nonautologous Frozen Red Cells into Peripheral Artery, Percutaneous Approach
ICD‐10‐PCS	30260H1	Transfusion of Nonautologous Whole Blood into Central Artery, Open Approach
ICD‐10‐PCS	30260N1	Transfusion of Nonautologous Red Blood Cells into Central Artery, Open Approach
ICD‐10‐PCS	30260P1	Transfusion of Nonautologous Frozen Red Cells into Central Artery, Open Approach
ICD‐10‐PCS	30263H1	Transfusion of Nonautologous Whole Blood into Central Artery, Percutaneous Approach
ICD‐10‐PCS	30263N1	Transfusion of Nonautologous Red Blood Cells into Central Artery, Percutaneous Approach
ICD‐10‐PCS	30263P1	Transfusion of Nonautologous Frozen Red Cells into Central Artery, Percutaneous Approach
CPT	36430	Transfusion, blood or blood components
HCPCS	P9010	Blood (whole), for transfusion, per unit
HCPCS	P9011	Blood, split unit (specify amount)
HCPCS	P9016	Red blood cells, leukocytes reduced, each unit
HCPCS	P9021	Red blood cells, each unit
HCPCS	P9022	Red blood cells, washed, each unit
HCPCS	P9038	Red blood cells, irradiated, each unit
HCPCS	P9039	Red blood cells, deglycerolized, each unit
HCPCS	P9040	Red blood cells, leukocytes reduced, irradiated, each unit
HCPCS	P9051	Whole blood or red blood cells, leukocytes reduced, cmv‐negative, each unit
HCPCS	P9054	Whole blood or red blood cells, leukocytes reduced, frozen, deglycerol, washed, each unit
HCPCS	P9056	Whole blood, leukocytes reduced, irradiated, each unit
HCPCS	P9057	Red blood cells, frozen/deglycerolized/washed, leukocytes reduced, irradiated, each unit
HCPCS	P9058	Red blood cells, leukocytes reduced, cmv‐negative, irradiated, each unit

Cardiac Tamponade/Perforation:

**Table:**

Code type	Code	Code description
ICD‐10‐CM	I31.2	Hemopericardium, not elsewhere classified
ICD‐10‐CM	I31.4	Cardiac tamponade
ICD‐10‐PCS	0W9C30Z	Drainage of Mediastinum with Drainage Device, Percutaneous Approach
ICD‐10‐PCS	0W9C3ZZ	Drainage of Mediastinum, Percutaneous Approach
ICD‐10‐PCS	0W9D30Z	Drainage of Pericardial Cavity with Drainage Device, Percutaneous Approach
ICD‐10‐PCS	0W9D3ZX	Drainage of Pericardial Cavity, Percutaneous Approach, Diagnostic
ICD‐10‐PCS	0W9D3ZZ	Drainage of Pericardial Cavity, Percutaneous Approach
ICD‐10‐PCS	0W9D40Z	Drainage of Pericardial Cavity with Drainage Device, Percutaneous Endoscopic Approach
ICD‐10‐PCS	0W9D4ZX	Drainage of Pericardial Cavity, Percutaneous Endoscopic Approach, Diagnostic
ICD‐10‐PCS	0W9D4ZZ	Drainage of Pericardial Cavity, Percutaneous Endoscopic Approach
HCPCS Code	G9408	Patients with cardiac tamponade and/or pericardiocentesis occurring within 30 days

Abbreviations: CPT, current procedural terminology; HCPCS, Healthcare Common Procedure Coding System ICD‐10, International Classification of Diseases, 10th revision; ICD‐9, International Classification of Diseases, 10th revision.
