# Supplementary material for: Differences in outcomes among patients with atrial fibrillation undergoing catheter ablation with versus without intracardiac echocardiography
Source: J Cardiovasc Electrophysiol. 2022 Jul 23;33(9):2015–47. doi: 10.1111/jce.15599 (PMC9544828; doi:10.1111/jce.15599)
Supplement: Supplementary file 1 — Supplementary information. [file JCE-33-2015-s001.docx]

**Supplemental Table 1.** Study population attrition for the assessment of complication outcomes.

| **Step** | **Attrition Step** | **N** |
| --- | --- | --- |
| 1 | Patients aged 18-64 years with catheter ablation (CA) procedure in an inpatient or outpatient setting with a primary diagnosis of atrial fibrillation (AF) between January 2016 and November 2020 (first such ablation considered as index ablation) | 25,501 |
| 2 | Include patients with continuous enrollment in the 12-month pre-index CA period | 19,924 |
| 3 | Exclude patients with CA (primary or secondary) procedure with a primary or secondary diagnosis of AF performed in the 12-month pre-index CA period | 19,672 |
| 4 | Exclude patients with Surgical ablation during the 12-month pre-index CA period | 19,545 |
| 5 | Exclude patients with Valvular procedure or atrioventricular node ablation during the 12-month pre-index CA period (including index ablation) | 19,397 |
| 6 | Exclude patients with left atrial appendage occlusion during the 12-month pre-index CA period (including index ablation) | 19,368 |
|  | **Total Sample**  **(ICE Use / No ICE Use)** | **19,368**  **(17,997 / 1,371)** |

AF: atrial fibrillation; CA: catheter ablation; ICE: intracardiac echocardiography

| **Step** | **Attrition Step** | **N** |
| --- | --- | --- |
| 1 | Patients aged 18-64 years with catheter ablation (CA) procedure in an inpatient or outpatient setting with a primary diagnosis of atrial fibrillation (AF) between January 2016 and December 2019 (first such ablation considered as index ablation) | 21,812 |
| 2 | Include patients with continuous enrollment in the 12-month pre-index CA period | 17,016 |
| 3 | Exclude patients with CA (primary or secondary) procedure with a primary or secondary diagnosis of AF performed in the 12-month pre-index CA period | 16,767 |
| 4 | Exclude patients with Surgical ablation during the 12-month pre-index CA period | 16,664 |
| 5 | Exclude patients with Valvular procedure or atrioventricular node ablation during the 12-month pre-index CA period (including index ablation) | 16,551 |
| 6 | Exclude patients with left atrial appendage occlusion during the 12-month pre-index CA period (including index ablation) | 16,524 |
|  | **Total Sample**  **(ICE Use / No ICE Use)** | **16,524**  **(15,274 / 1,250)** |

**Supplemental Table 2.** Study population attrition for the assessment of 12-month healthcare utilization outcomes.

AF: atrial fibrillation; CA: catheter ablation; ICE: intracardiac echocardiography
